# A structural intermediate pre-organizes the *add* adenine riboswitch for ligand recognition

**DOI:** 10.1093/nar/gkab307

**Published:** 2021-05-08

**Authors:** Patrick St-Pierre, Euan Shaw, Samuel Jacques, Paul A Dalgarno, Cibran Perez-Gonzalez, Frédéric Picard-Jean, J Carlos Penedo, Daniel A Lafontaine

**Affiliations:** Department of Biology, Faculty of Science, Université de Sherbrooke, Sherbrooke, Quebec J1K 2R1, Canada; Centre of Biophotonics, School of Physics and Astronomy, University of St Andrews, St Andrews, Fife, KY16 9SS, UK; Department of Biology, Faculty of Science, Université de Sherbrooke, Sherbrooke, Quebec J1K 2R1, Canada; Centre of Biophotonics, School of Physics and Astronomy, University of St Andrews, St Andrews, Fife, KY16 9SS, UK; Biomedical Sciences Research Complex, University of St Andrews, St Andrews, Fife, KY16 9SS, UK; Department of Biology, Faculty of Science, Université de Sherbrooke, Sherbrooke, Quebec J1K 2R1, Canada; Centre of Biophotonics, School of Physics and Astronomy, University of St Andrews, St Andrews, Fife, KY16 9SS, UK; Biomedical Sciences Research Complex, University of St Andrews, St Andrews, Fife, KY16 9SS, UK; Department of Biology, Faculty of Science, Université de Sherbrooke, Sherbrooke, Quebec J1K 2R1, Canada

## Abstract

Riboswitches are RNA sequences that regulate gene expression by undergoing structural changes upon the specific binding of cellular metabolites. Crystal structures of purine-sensing riboswitches have revealed an intricate network of interactions surrounding the ligand in the bound complex. The mechanistic details about how the aptamer folding pathway is involved in the formation of the metabolite binding site have been previously shown to be highly important for the riboswitch regulatory activity. Here, a combination of single-molecule FRET and SHAPE assays have been used to characterize the folding pathway of the adenine riboswitch from *Vibrio vulnificus*. Experimental evidences suggest a folding process characterized by the presence of a structural intermediate involved in ligand recognition. This intermediate state acts as an open conformation to ensure ligand accessibility to the aptamer and folds into a structure nearly identical to the ligand-bound complex through a series of structural changes. This study demonstrates that the *add* riboswitch relies on the folding of a structural intermediate that pre-organizes the aptamer global structure and the ligand binding site to allow efficient metabolite sensing and riboswitch genetic regulation.

## INTRODUCTION

Gene expression requires the assistance of numerous cofactors to provide the specificity and timing of regulation, which are important to ensure cellular homeostasis. Accumulating evidence suggests that RNA folding is central for gene regulation, particularly by providing specific protein binding sites and important architectural domains (1). A striking example about the importance of RNA folding in gene expression was obtained with the discovery of riboswitches that are genetic regulatory elements found in the 5′ untranslated region (5′ UTR) of bacterial messenger RNA (mRNA) (2–4). Riboswitches are involved in the control of gene expression by modulating their structure upon the binding of specific cellular metabolites (2). The recognition of the metabolite is mediated by the highly conserved aptamer domain that exhibits a high degree of structural complexity (5). The formation of the aptamer-ligand complex influences the structure of the riboswitch expression platform that is directly implicated in the regulation of gene expression, either by modulating mRNA levels or the initiation of translation (2,4). Riboswitches have also been involved to modulate splicing (6,7) and to regulate gene expression in a *trans*-acting manner (8).

**Figure 1.**
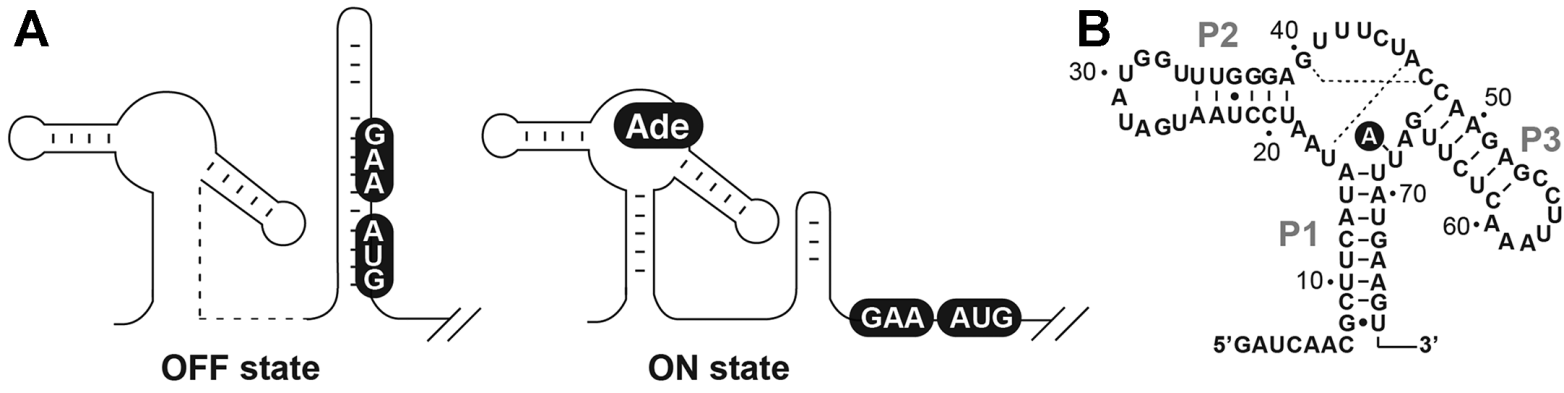
Regulatory scheme of the *Vibrio vulnificus add* adenine riboswitch. (**A**) Secondary structure model of the ON and OFF conformers of the adenine riboswitch. In the absence of adenine, the riboswitch allows the formation of a stem-loop sequestering the ribosome access to the Shine-Dalgarno (GAA) and AUG initiation codon (boxed), thus preventing the initiation of translation. However, upon adenine (Ade) binding, the riboswitch folds into an alternative structure in which the Shine-Dalgarno and the AUG initiation codon are accessible to allow translation initiation. (**B**) Secondary structure model of the *add* adenine aptamer. The sequence and secondary structure are shown with P1, P2 and P3 helical domains. Dotted lines represent Watson–Crick base pairing interactions occurring within the aptamer core region. The bound adenine is shown in a black circle. The numbering is based from a previous study (66).

Riboswitches recognize a large variety of metabolites such as amino acids, nucleobases and cofactors (3,9). Purine-sensing riboswitches are among the most characterized riboswitch classes and are organized around a single three-way junction, making them particularly tractable for structure-function analyses (10–19). Purine riboswitches can be categorized in four natural groups that respectively bind to adenine (20), guanine (21), deoxyguanosine (22) and 7-aminomethyl-7-deazaguanine (preQ1) (23). The adenine-sensing *Vibrio vulnificus add* riboswitch controls the initiation of translation of an adenine deaminase (*add*) upon ligand binding (Figure 1A), therefore making it one of the rare riboswitches to positively modulate gene expression upon ligand sensing (3,4). In the absence of adenine, the formation of a stem-loop structure sequesters the Shine-Dalgarno (SD) and AUG start codon sequences to inhibit translation initiation (Figure 1A, OFF state). Upon ligand binding, the structure of the riboswitch is reorganized to allow ribosome access, thus inducing translation initiation (Figure 1A, ON state). The *add* aptamer is composed of three helical domains (stems P1, P2 and P3) that are connected through a highly conserved core domain comprising the ligand binding site (Figure 1B) (20). Crystal structures of ligand-bound adenine and guanine aptamers (24,25) have revealed that they share a similar 3D structure in which the RNA is very compact and contains several RNA ligand contacts (26). The structure of the metabolite-RNA complex exhibits a long-range loop loop tertiary interaction occurring between loops L2 and L3 that is crucial for ligand binding (27). The P1 and P3 stems are involved in a coaxial stack, a feature often present in three-way helical domains (28–31). The ligand binding site is constituted by a cavity in which the bound metabolite is sandwiched between conserved residues and is involved in interactions with specific nucleotides (24,25). Of particular importance is residue U68 (Figure 1A) that is involved in a Watson-Crick base pair with the bound adenine. The substitution of U68 for a cytosine converts the specificity toward guanine (20). The ligand binding specificity is also ensured by multiple interactions such as Watson–Crick base pairs (G40:C47 and U16:A46) participating in base triple interactions (24,25). In addition to the ligand-bound structure, a recent analysis performed with femtosecond X-ray free electron laser (XFEL) methods revealed that the adenine aptamer can sample at least four structural conformations including two ligand-free and two ligand-bound states (15). Notably, while all XFEL aptamer structures contain the long-range L2–L3 tertiary interaction, they show different molecular interactions in the core region suggesting that the ligand-binding site is structurally flexible.

The folding mechanism of both the adenine and guanine aptamers have been previously characterized using several biochemical and biophysical techniques (11,32,41,33–40). While both aptamers share a common ligand-bound structure, it appears that they do not rely on similar strategies to adopt the ligand-bound native state. This is supported by the fact that the adenine-sensing aptamer exhibits a larger degree of structural flexibility compared to the guanine-sensing variant (40,42–45). Previously, single-molecule Förster resonance energy transfer (smFRET) assays have been used to study the folding of the *add* riboswitch aptamer (27,46,47). Real-time smFRET traces revealed that ligand-free aptamers fluctuate between three different states (unfolded (U), intermediate (I), and folded (F)) and exhibit a high degree of dynamic heterogeneity that is significantly reduced upon ligand binding (27,43). Importantly, the L2–L3 interaction was suggested to be absent in the intermediate state (27), therefore indicating that it may represent a different structure than those identified using XFEL (15). The absence of the L2–L3 interaction in the intermediate state suggests that this conformer appears early during the riboswitch folding pathway and that it could be important for the formation of the ligand binding site (27).

Here, smFRET assays were used to characterize the nature of the intermediate state by monitoring several intramolecular distances of the *add* aptamer and their relative variation along the folding pathway. The results are consistent with the intermediate conformer corresponding to a partially folded aptamer in which the P2 stem has not yet attained the position observed in crystal structures (24,25). We determined that the intermediate structure is stabilized when preventing the formation of the L2–L3 loop–loop interaction or when adding sub-saturating concentrations of a denaturing agent such as urea (12). Furthermore, the data showed that the P2 stem is the most flexible domain of the aptamer and that it is rotated toward the P3 stem from the unfolded to the folded state at which point it can interact with the P3 stem to promote the L2–L3 interaction. smFRET analysis revealed that multiple conformations of the core region are modulated by both the formation of the L2–L3 loop–loop interaction and ligand binding, suggesting that the core region may freely sample various folds across different aptamer global structures. Lastly, biochemical probing of an *add* aptamer stabilized in the intermediate state revealed that the U16:A46 base pair is not yet formed, suggesting a pre-folded architecture. Together, these results indicate that the intermediate conformer represents a structural scaffold that allows ligand biding to the aptamer domain prior to the formation of the L2–L3 loop–loop interaction, which is required to modulate gene expression.

## MATERIALS AND METHODS

### Preparation of dual-labeled aptamer molecules.

Fluorescent adenine aptamers were assembled as previously reported (27). Briefly, aptamer molecules were reconstituted by ligating two RNA molecules each carrying a Cy3 or Cy5 fluorophore (see Supplementary Table S2 for RNA sequences). The RNA strand corresponding to the 5′ portion of the *add* aptamer carried a biotin at the 5′ end to allow surface attachment and a 5-amino-allyl uridine nucleotide for specific Cy5 labeling. The RNA strand corresponding to the aptamer 3′ portion carried a phosphate group at the 5′ extremity to allow subsequent RNA ligation and a 5-amino-allyl uridine nucleotide for specific Cy3 labeling. RNA strands were labeled using NHS ester fluorophores (Fisher Scientific). T4 RNA ligation and purification of RNA strands were performed as previously described (27). Briefly, purified Cy3- and Cy5-labeled RNA strands (1:1 ratio) were annealed in 10 mM HEPES (pH 7.5), 50 mM NaCl and slowly cooled from 80°C to room temperature. T4 RNA ligase (New England BioLabs) was added to the reaction and samples were incubated for 4 hours at 37°C. Ligated RNA molecules were isolated using denaturing polyacrylamide gel electrophoresis and electroelution.

### Single-molecule FRET analysis.

smFRET analysis were performed as previously reported (27). Briefly, molecules were isolated on quartz microscope slides using successive treatment with 1 mg/ml biotinylated BSA (Sigma) and 0.2 mg/ml streptavidin (Invitrogen). After treatment with streptavidin, 50–250 pM of fluorophore-labeled aptamers were added to the slide. Fluorescence data at donor and acceptor wavelengths were acquired from single molecules using a total internal fluorescence (TIR) microscope coupled to an EMCCD camera (iXon, Andor Technology). Data were obtained with an integration time of 50 ms unless stated otherwise. The imaging buffer used for all experiments contained 10 mM Tris–HCl pH 8.0, 10 mM NaCl, 1 μM protocatechuic acid (Fisher Scientific), 0.25 μM protocatechuic dioxygenase (Sigma) and 0.5 mM 6-hydroxy-2,5,7,8-tetramethyl-chroman-2-carboxylic acid (TROLOX) (Sigma) (48). Where indicated, a concentration of 10 mM magnesium and 1 mM 2,6-diaminopurine (DAP, Sigma) was directly added to the imaging buffer. Single-molecule FRET efficiency (*E*_FRET_) after background correction was determined by: *E*_FRET_ = *I*_A_/(*I*_A_ + *I*_D_) where *I*_A_ and *I*_D_ are the fluorescence intensities of Cy5 and Cy3, respectively. Data analysis was made using custom-written analysis routine developed in IDL 6.0 and MATLAB 7 (27). The smFRET histograms were built by extracting for each trace the average FRET efficiency of the first 10 frames of the single-molecule trajectory as previously reported (27). An exception to this procedure was made for the smFRET histograms of vector P1–L3 (Figure 4). Given the proximity of *E*_FRET_ values obtained across experimental conditions, the smFRET histograms for this vector were generated using a cumulative-FRET approach where the first 30 s of each FRET trace were included in the analysis. Statistical significance of mean FRET values for each experimental condition was evaluated using unpaired t-test methods. Idealized single-molecule trajectories were obtained using Hidden Markov Modelling (HMM) implemented in a software package (Hammy) available from the Single-Molecule Nanometry Group at the University of Illinois at Urbana-Champaign (USA) as previously described (49). Briefly, Hammy treats a single-molecule trajectory as a Markov process with an unknown (‘hidden’) number of states. At each step in an ideal Markov process, a transition between the current state and any other can happen with a characteristic probability, and the system has no memory of previous states. The Hammy algorithm models an input trajectory as a Markov process with the appropriate number of states and optimized transition probabilities between them. The transition probabilities (fitted in units of transitions per frame) can be linked to average transition states by multiplying by the frame rate. Single-molecule dwell-time information as a function of urea and NaCl concentration was obtained using two-state HMM unless stated otherwise. Single-molecule dwell-time histograms were fitted to mono-exponential decay functions to extract the folding and unfolding rates.

### SHAPE chemical probing of adenine aptamers.

Transcripts corresponding to the wild-type and L2 mutant aptamers were produced as previously described (50,51). Briefly, SHAPE reactions were prepared by mixing 1 pmol of purified aptamers resuspended in a 2:1 mixture of 0.5X TE buffer: 3.3X folding buffer (333 mM K-HEPES, pH 8.0, 333 mM NaCl) and the required concentration of MgCl_2_ and DAP. Samples were heated to 65°C and allowed to cool slowly at room temperature before being preincubated 10 min at 37°C. Samples were reacted in 13 mM *N*-methylisatoic anhydride (NMIA, Invitrogen) dissolved in dimethyl sulfoxide for 80 min at 37°C (50). The modified RNA was ethanol precipitated, washed with 70% ethanol and resuspended in 0.5× TE buffer. Reverse transcription reactions were performed on NMIA-reacted RNA and products were separated on 5% denaturing polyacrylamide gels.

The quantification was performed using the software Quantity One 4.6.6 (Bio-Rad) where the volume function was employed to quantify the intensity of each band of interest. An empty region of the gel was used for background subtraction. The intensity of each band was normalized using position 59 that was not modulated across tested conditions. The 0 mM MgCl_2_ data of the wild-type and L2 mutant experiments were used for normalization and were thus arbitrarily reported to a value of 1. Experiments have been done at least in duplicate.

## RESULTS

### smFRET analysis of the folding pathway of the *add* aptamer.

The ability of smFRET to resolve transient RNA structures and to determine intramolecular distances was used to characterize the structural features of intermediate state and its role in metabolite recognition. The ligand-induced folding of the aptamer was assessed using 2,6-diaminopurine (DAP) since crystal structures showed that DAP and adenine binding produce RNA–ligand complexes having very similar structures (24,39). Importantly, DAP exhibits a ∼30-fold affinity increase toward the adenine aptamer (20), which is caused by an additional hydrogen bond formed between DAP and U68 (39). These results confirm that DAP can be used to study the folding of *add* aptamers in the context of the wild-type but also using informative low-affinity structural mutants.

**Figure 2.**
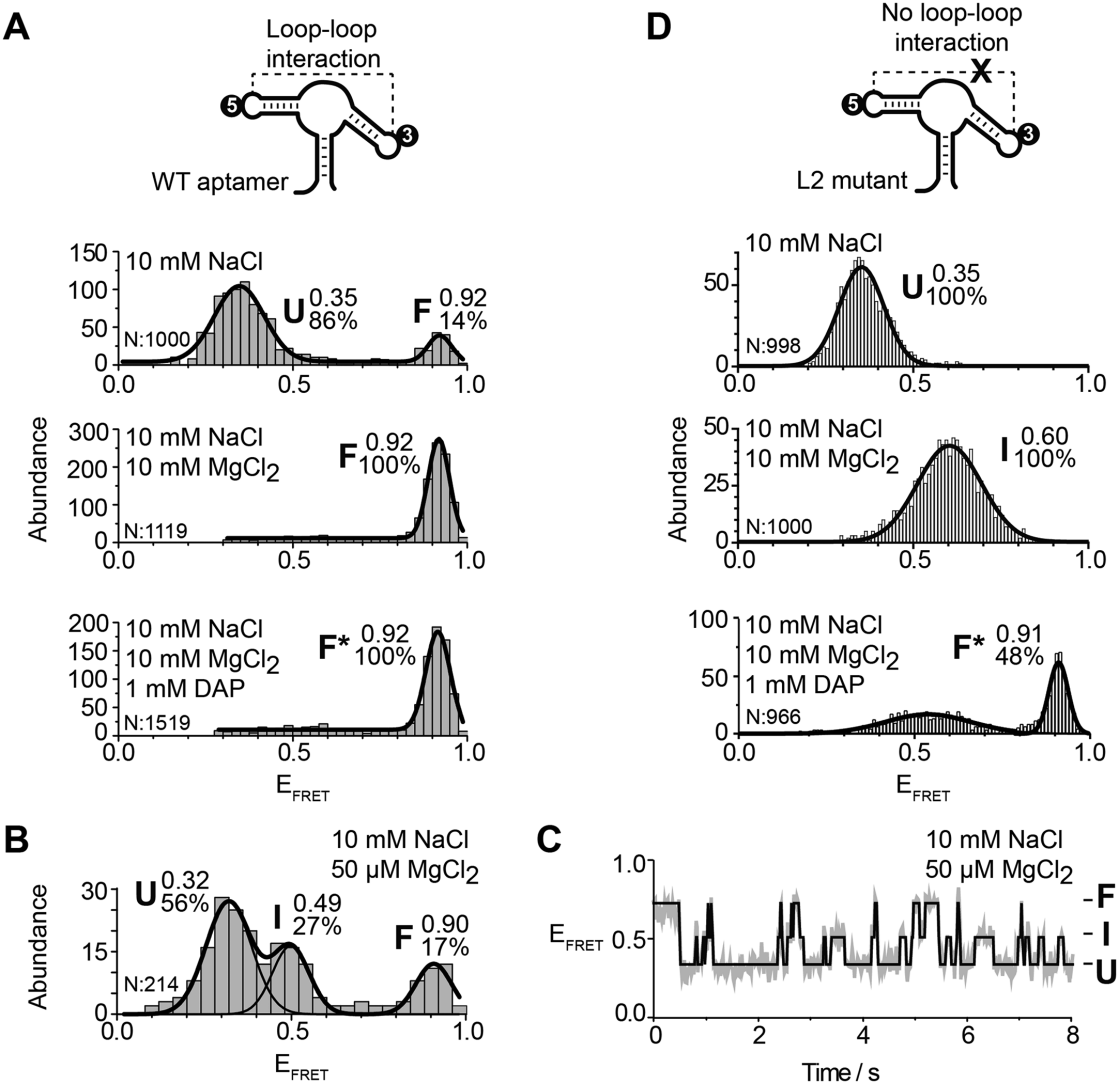
smFRET analysis for the vector P2–P3 of the adenine aptamer. (**A**, **D**) smFRET experiments performed for the wild-type (A) and the L2 mutant (D) aptamers. The cartoon represents the dual-labeled aptamer used in each case. The labeling positions of the donor Cy3 (3) and the acceptor Cy5 (5) are indicated. Population histograms are shown in presence of 10 mM NaCl (top panel), 10 mM NaCl and 10 mM MgCl_2_ (middle panel) and 10 mM NaCl, 10 mM MgCl_2_ and 1 mM DAP (lower panel). E_FRET_ values determined by fitting analysis to a Gaussian distribution for each population are indicated. Unfolded (U), intermediate (I), ligand-free folded (F) and ligand-bound folded (F*) are shown. The number of analyzed molecules (N) and the proportion of each analyzed population are indicated. (**B**, **C**) smFRET histograms (B) and a time trace (C) for the wild-type aptamer in 10 mM NaCl and 50 μM MgCl_2_. The corresponding FRET structural states are shown at the right of the time trace. The solid line represents the three-state trajectory obtained from a hidden Markov modeling of the experimental trace.

To obtain Cy3-Cy5 dual-labeled aptamers to be used in smFRET assays, we have previously engineered the *add* aptamer to have a more stable P1 stem (27). The fluorescent aptamer was designed to be reconstituted from two RNA strands that are ligated by the T4 RNA ligase. For the current study, this construct will be referred to as the wild-type sequence. Using this strategy, we first attached fluorophores in the aptamer L2 and L3 loops (L2–L3 vector) to report the formation of the loop–loop interaction (Figure 2A, see scheme) (27,43). As previously observed, in conditions in which the loop–loop interaction is not formed (10 mM NaCl), a large proportion of aptamers adopted the U state (*E*_FRET_ ∼ 0.35) (Figure 2A, top panel and see Supplementary Table S1 for fitted smFRET parameters) (27). A small fraction of the aptamer population also folded in the F state in which the loop–loop interaction is expected to occur (*E*_FRET_ ∼ 0.92). The addition of Mg^2+^ ions shifted the majority of aptamers to the F state (Figure 2A, middle panel), consistent with the loop–loop interaction being stabilized with Mg^2+^ ions, as previously observed (27). In the presence of DAP, *add* aptamers adopted a conformation exhibiting a FRET value identical to the F state (*E*_FRET_ ∼ 0.92) (Figure 2A, bottom panel), suggesting that ligand binding does not rearrange the loop–loop interaction to a detectable level. Because ligand-free and ligand-bound structures are structurally different at the level of the aptamer core region (15,20), the ligand-bound was named F* to distinguish it from the ligand-free F state.

We previously reported that the *add* aptamer folded through the I state and that this conformer was more populated at low magnesium ions concentrations (27). To detect the presence of the I state, *add* aptamers were incubated in a buffer containing 50 μM MgCl_2_. As previously observed, aptamer molecules were found to adopt the U, I and F states (Figure 2B). Analysis of time traces showed that single aptamers transited between the three states (Figure 2C), consistent with the I state being frequently sampled during the folding process.

### Disruption of the L2–L3 interaction stabilizes the formation of the intermediate state.

The intermediate conformer of the *add* aptamer might correspond to a structure in which the L2–L3 loop–loop interaction is not yet formed (27). The L2–L3 interaction relies on the formation of five base pairs (24,25). Of these interactions, two pairs (G31–C55 and G32–C54) correspond to Watson–Crick base interactions and the remaining three are involved in *trans* base pairs. In an attempt to block the folding of the intermediate structure into the F state, we engineered a L2 mutant aptamer (U28A, A29U, G31C and G32C) to disable the L2–L3 loop–loop interaction.

smFRET analysis of the L2 mutant showed a U state very similar to the wild-type in the presence of NaCl (Figure 2D, top panel). Importantly, the L2 mutant exhibited a very different FRET value in Mg^2+^ ions (*E*_FRET_ ∼ 0.60) compared to the wild type (Figure 2D, middle panel). This FRET value is similar to the value observed in the context of the wild type at low concentrations of ions and ligand (Figure 2B) (27). Titration of the Mg^2+^ concentration revealed that the I state was progressively favored at high Mg^2+^ concentrations, consistent with the I state being stabilized by the binding of Mg^2+^ ions (Supplementary Figure S1). The folding of the L2 mutant was also monitored in the presence of DAP. Strikingly, the addition of DAP resulted in *add* aptamers folding into the F* state (Figure 2D, bottom panel). Therefore, although the L2 and L3 loop sequences are not compatible to interact in the L2 mutant, the high FRET value (∼0.91) of the F* state suggests that they are in very close proximity (Figure 2D, lower panel). Control experiments showed that the F* state could be obtained in different proportions by varying the concentration of adenine or when using a smaller concentration of DAP (Supplementary Figure S2), indicating that the F* state represents the formation of a ligand-aptamer complex. The U, I and F* states were also observed using a L3 mutant (Supplementary Figure S3), suggesting that the identity of loop sequences is not important for the ligand-induced F* state. Lastly, we also investigated whether the DAP-dependent aptamer folding required the presence of MgCl_2_ in the context of a disabled loop–loop interaction (L2 mutant). When assessing the folding of the L2–L3 vector in the absence of Mg^2+^ ions, but with DAP, aptamer molecules adopted the U state (Supplementary Figure S4), suggesting that Mg^2+^ ions are required for the formation of the DAP-L2 mutant complex.

The importance of the L2–L3 interaction for the I state was also studied in a mutant aptamer bearing G31C and G32C mutations, thereby only preventing the formation of L2–L3 Watson–Crick base pairs. Similarly to the L2 or L3 mutant, the FRET value increased in the presence of Mg^2+^ ions (∼0.42) and DAP (∼0.89), suggesting that the I and F* states were adopted, respectively (Supplementary Figure S5). Our results show that the introduction of only two mutations preventing Watson-Crick pairing is sufficient to inhibit the formation of the L2–L3 interaction in a background of Mg^2+^ ion concentrations, in agreement with previous studies (27,52,53). Thus, smFRET analysis of the L2–L3 loop–loop interaction revealed that the I state of the *add* aptamer is stabilized by preventing the formation of the loop–loop interaction. However, DAP binding to the intermediate conformer allows stems P2 and P3 to be brought into close proximity in both wild-type and mutant aptamers.

### Probing the intermediate state using the competing interplay between folding and unfolding agents

We showed that disruption of the loop–loop interaction blocks the folding pathway of the *add* aptamer resulting in a single population of aptamer mutants with a FRET value similar to that of the I state. However, in the context of the wild-type aptamer, the intermediate state is intrinsically a short-lived and low-populated state, and therefore, only represents a small proportion with respect to the folded (F and F*) and U states. To further confirm that all wild-type aptamer molecules are indeed able to adopt the I state, we designed a new experimental approach to overpopulate the intermediate state. We previously showed that urea-induced RNA denaturation can be used to estimate the relative stabilization of the F* state, with respect to F, induced by ligand binding (12,41). It has been also demonstrated that although F and F* have identical FRET values, both states exhibit a remarkably different urea-induced unfolding rate and this kinetic feature can be used to unambiguously differentiate them (12). Based on those findings, we hypothesized that by balancing the concentrations of folding and denaturant agents, it should be possible to influence the aptamer kinetics to study the intermediate state. Ideally, the folding agent should exclusively promote transitions into the folded state and the denaturant agent should only act on the reverse pathway. It has been previously shown that Mg^2+^ ions not only accelerate folding but also slow down the unfolding rate (12), therefore instead of using a combination of Mg^2+^ ions and urea, we used Na^+^ ions as potential folding agents.

**Figure 3.**
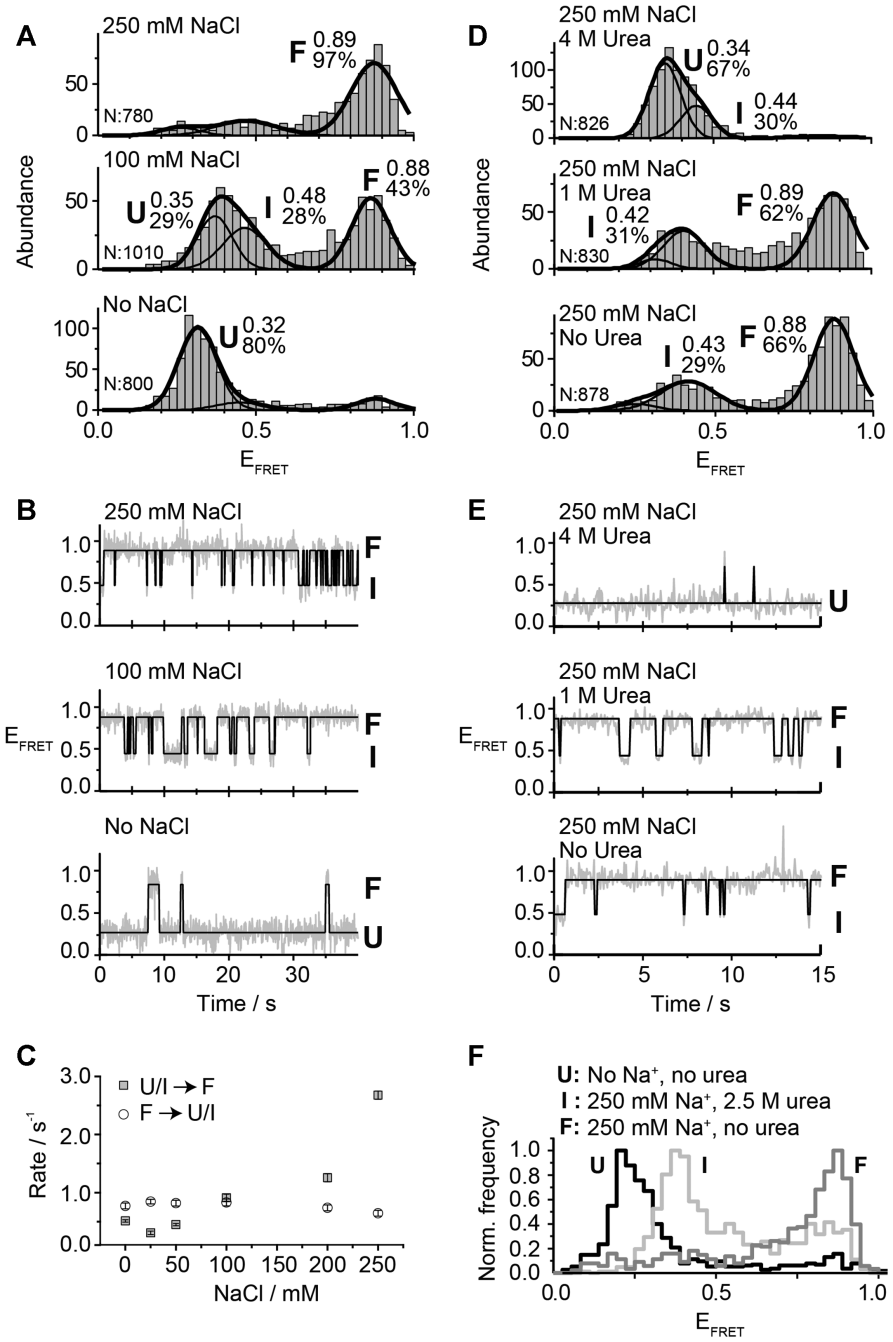
smFRET analysis for the vector P2–P3 as a function of NaCl and urea. (**A**, **B**) smFRET histograms (A) and time traces (B) experiments performed for the wild-type aptamer as a function of NaCl concentration. The NaCl concentration used in each experiment is indicated above each graph. The number of analyzed molecules (N) and the proportion of each analyzed population are indicated. (**C**) Effect of NaCl on the transition rates for the folding process from U/I to F states (grey squares) and for the unfolding process from F to U/I states (empty circles). (**D**, **E**) smFRET histograms (D) and time traces (E) experiments performed for the wild-type aptamer in 250 mM NaCl as a function of urea concentrations. The urea concentration used in each experiment is indicated above each graph. E_FRET_ values determined by fitting analysis to a Gaussian distribution for each population are indicated. The solid line represents the three-state trajectory obtained from a hidden Markov modeling of the experimental trace. (**F**) Normalized single-molecule FRET histograms obtained with no NaCl added (dark grey, U state), in a background of 250 mM NaCl and 2.5 M urea (light grey, I state) and with 250 mM NaCl and no urea (grey, F state).

Because the folding mechanism of the *add* aptamer has not been studied in depth with Na^+^ ions, we decided to carry out a titration of Na^+^ ions to determine the folding efficiency of monovalent ions to obtain the folded state (Figure 3A). We observed that as the concentration of Na^+^ ions was increased, a progressive shift from the U (*E*_FRET_ ∼ 0.32) to the F state (*E*_FRET_ ∼ 0.89) occurred, the latter becoming the predominant population above 100 mM Na^+^. The I state (*E*_FRET_ ∼ 0.48) was also observed and reached a maximum contribution at 100 mM Na^+^ ions (Figure 3A). smFRET trajectories displayed transitions from U, I and F states and the frequency of these events was higher as the concentration of Na^+^ ions was progressively increased (Figure 3B). Single-molecule kinetic analysis revealed a significant increase (∼10-fold) in the folding rate (U/I states → F state) from a value of 0.19 ± 0.02 s^−1^ without the addition of Na^+^ ions to a value of 2.7 ± 0.1 s^−1^ at 250 mM Na^+^ ions (Figure 3C). In contrast, the unfolding rate (F state → U/I states) was almost unchanged from 0.84 ± 0.03 s^−1^ at 0 mM NaCl to 0.64 ± 0.07 s^−1^ at 250 mM Na^+^ (Supplementary Figure S6A).

Since Na^+^ ions affect mostly the folding rate but have no effect on the unfolding rate, these conditions constitute an ideal environment to test the ability of urea to shift the folding equilibrium towards the I state. To test this, an urea titration was performed in a background of 250 mM Na^+^ ions, which promotes almost exclusively the F state (Figure 3D). As the urea concentration was increased, the F state progressively disappeared in favor of the U and I conformers (Figures 3D and 3E). At 2.5 M concentration of urea, a very small proportion of the U state was observed and the relative contribution of the I state (*E*_FRET_ ∼ 0.45) reached a value of 78% (Figure 3F and Supplementary Figure S6B). At higher urea concentrations only aptamers in the U and I states were observed (Figures 3D and 3E). A cross-correlation analysis of the smFRET trajectories obtained for aptamer molecules in the I state lasting for tens of seconds did not show evidence of hidden dynamics, suggesting that at least within a 50 ms time resolution these aptamers are locked in the intermediate conformation (Supplementary Figure S6C).

Next, we took advantage of the fact that specific ratios of folding and unfolding agents allow to selectively place the *add* aptamer into the U, I or F states to demonstrate that the I state is an on-path conformer. Using a 3M urea and 200 mM NaCl combination, we found that *add* aptamers populated only the U or I states. Because no folded conformation was detected in the absence of ligand (Supplementary Figure S7, top panel), such combination of urea and NaCl provides an opportunity to prove that the I state can recognize the ligand and form the F* state, thus unambiguously confirming its on-path character. As we increased the concentration of adenine ligand, we observed an increase in the population of F* and a parallel decrease in the relative populations of U and I (Supplementary Figure S7). Importantly, the obtained single-molecule FRET trajectories showed clear transitions between all states including direct transitions from the I state to the high-FRET state (F*) representing the ligand-bound folded state (Supplementary Figure S8), thus supporting the assignation of the I state as a ligand-binding competent structure. Single-molecule FRET trajectories showing transitions between three states were also obtained at these conditions (Supplementary Figure S8). As previously reported for three-state trajectories obtained at low concentration of Mg^2+^ ions (∼20 μM) (54), the formation of the F* state takes place almost exclusively from the I conformer.

Taken together, these results unambiguously demonstrate that the I state structurally emerges as an on-path conformer during the folding of the *add* aptamer. Our experiments also provide a clear example of how harnessing the competing action of folding and unfolding agents constitutes a useful approach to manipulate RNA folding mechanisms, and as observed for the *add* aptamer, to demonstrate the existence of otherwise difficult to capture transient states (Figure 3F).

### The P1–P3 helical stack is a relatively rigid structural scaffold.

**Figure 4.**
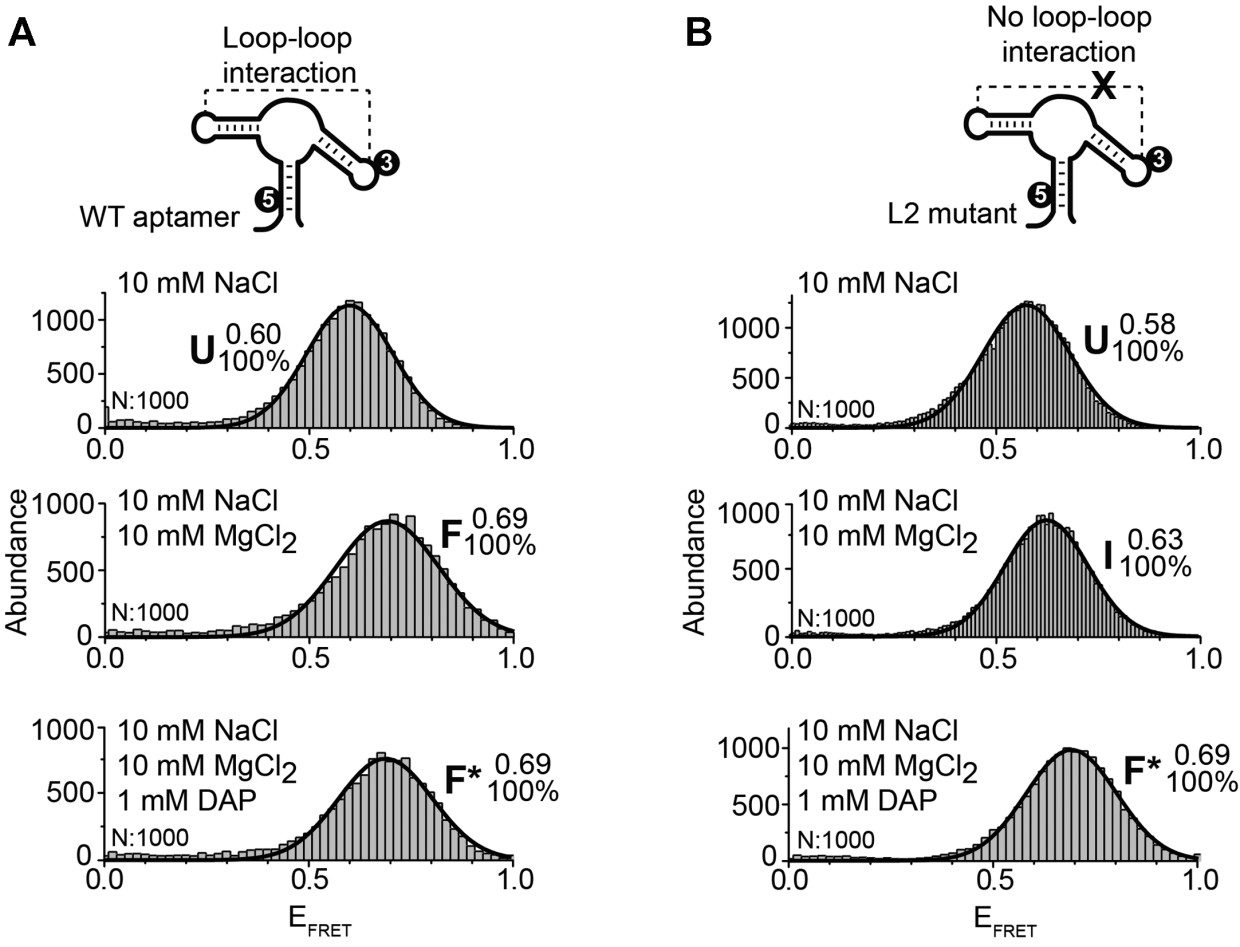
smFRET analysis for the vector P1–P3 of the adenine aptamer. (**A**, **B**) smFRET experiments performed for the wild-type (A) and the L2 mutant (B) aptamers. The cartoon represents the dual-labeled aptamer used in each case. Cy3 (3) and Cy5 (5) fluorophores are indicated. Population histograms are shown in presence of 10 mM NaCl (top panel), 10 mM NaCl and 10 mM MgCl_2_ (middle panel) and 10 mM NaCl, 10 mM MgCl_2_ and 1 mM DAP (lower panel). *E*_FRET_ values determined by fitting analysis for each population are indicated. Unfolded (U), intermediate (I), ligand-free folded (F) and ligand-bound folded (F*) are shown. The number of analyzed molecules (N) and the proportion of each analyzed population are indicated.

To gain additional knowledge about the structural organization of the I state, smFRET studies using a P1–P3 distance vector were carried out (Figure 4A, see scheme) (24). The wild-type aptamer was found in a single population (U state, *E*_FRET_ ∼ 0.60) in the presence of NaCl (Figure 4A, top panel). The addition of Mg^2+^ ions yielded a minor but reproducible FRET change (F state, *E*_FRET_ ∼ 0.69) (Figure 4A, middle panel), suggesting a small structural change upon the binding of Mg^2+^ ions. No FRET change was observed upon the addition of DAP (Figure 4A, bottom panel). In the context of the L2 mutant (Figure 4B), the FRET efficiencies obtained for the U and F* states were identical to those reported for the wild-type construct, but an additional ligand-free intermediate state (*E*_FRET_ ∼ 0.63) was detected in the presence of Mg^2+^ ions (Figure 4B, middle panel). The statistical significance of this value compared to the *E*_FRET_ value of U and F was confirmed using *t*-test analysis (*P* < 0.0001) (Supplementary Figure S9A). Moreover, a cross-correlation analysis of the smFRET trajectories obtained for this intermediate revealed not hidden dynamics within our time resolution (100 ms), suggesting this mutant-specific state does not result from the averaging of fast interconversion between states U and F (Supplementary Figure S9B). Due to the relatively small changes in *E*_FRET_ values observed across experimental conditions (Figure 4), the P1–P3 stacking interaction is most likely formed within each state. The relative lack of structural changes involving the P1–P3 helical domain is in good agreement with similar observations made with the guanine aptamer (43).

### The P1–P2 inter-helical distance is highly dependent on cofactors binding.

**Figure 5.**
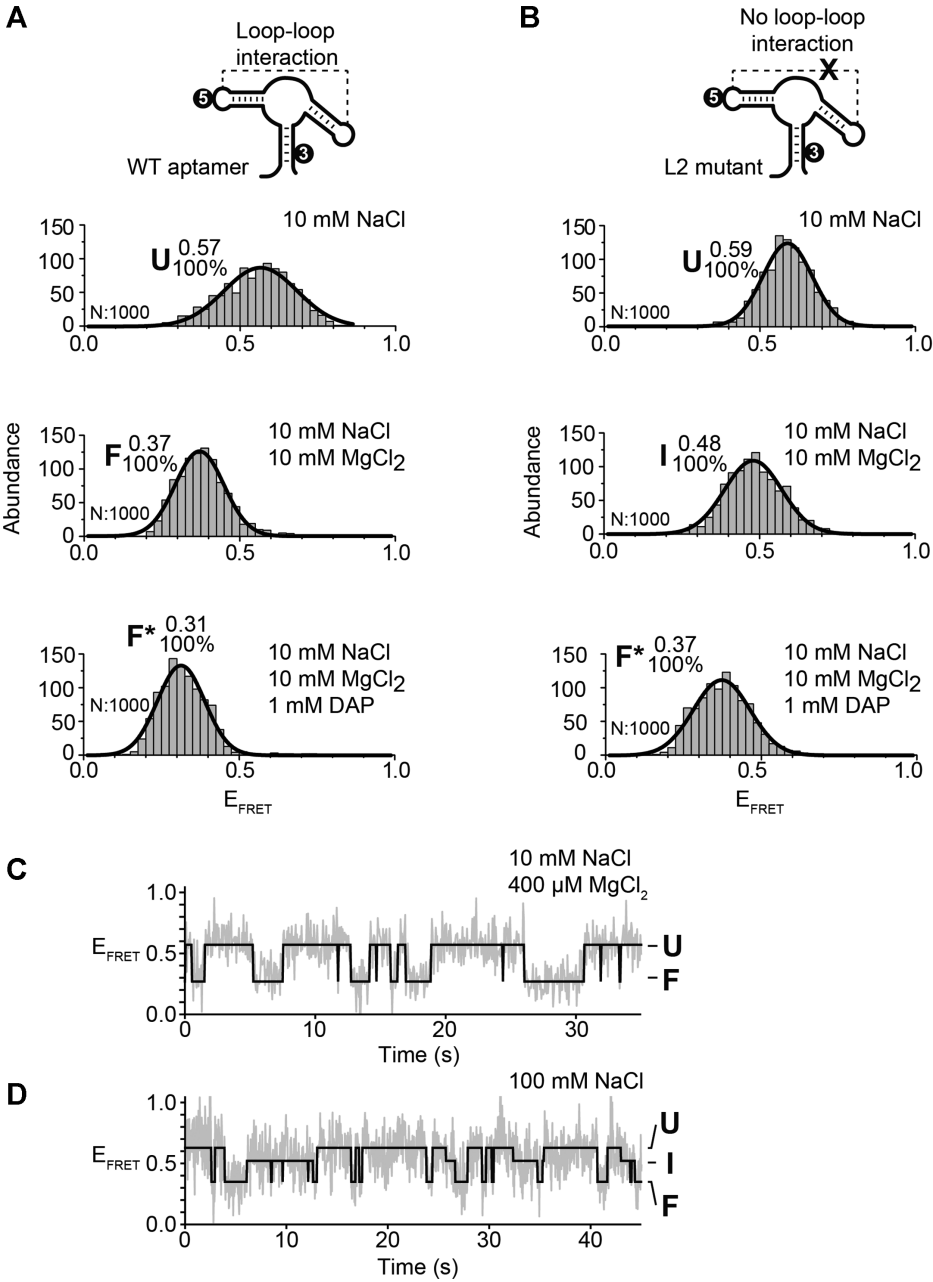
smFRET analysis for the vector P1–P2 of the adenine aptamer. (**A**, **B**) smFRET experiments performed for the wild-type (A) and the L2 mutant (B) aptamers. The cartoon represents the dual-labeled aptamer used in each case. Cy3 (3) and Cy5 (5) fluorophores are indicated. Population histograms are shown in presence of 10 mM NaCl (top panel), 10 mM NaCl and 10 mM MgCl_2_ (middle panel) and 10 mM NaCl, 10 mM MgCl_2_ and 1 mM DAP (lower panel). E_FRET_ values determined by fitting analysis for each population are indicated. Unfolded (U), intermediate (I), ligand-free folded (F) and ligand-bound folded (F*) are shown. The number of analyzed molecules (N) and the proportion of each analyzed population are indicated. (**C**, **D**) Time traces in 10 mM NaCl and 400 μM MgCl_2_ (top panel) and in 100 mM NaCl (bottom panel). The corresponding FRET structural states are shown at the right of the time trace. The solid line represents the three-state trajectory obtained from a hidden Markov modeling of the experimental trace.

The relatively invariant conformation of the P1–P3 stacking unit (Figure 4A) suggests that the large structural changes observed for the L2–L3 interaction (Figure 2A) are mostly caused by the repositioning of the P2 helix relative to the P1–P3 helical stack. To monitor relative movements of the P2 stem, FRET fluorophores pairs were positioned to detect the distance changes occurring between stems P1 and P2. A single aptamer population was observed in the presence of 10 mM NaCl (U state, *E*_FRET_ ∼ 0.57) (Figure 5A, top panel). We detected a significant decrease in the P1–P2 interhelical distance upon the addition of both Mg^2+^ ions (F state, *E*_FRET_ ∼ 0.37) and DAP (F* state, *E*_FRET_ ∼ 0.31) (Figure 5A, middle and bottom panels), suggesting that both cofactors modulate the distance between P1 and P2 stems.

In the context of the L2 mutant aptamer, the addition of NaCl yielded a single U population very similar to the wild-type aptamer (Figure 5B, top panel). Furthermore, the addition of Mg^2+^ ions induced the formation of the I state having a FRET value of ∼0.48 (Figure 5B, middle panel). Importantly, this FRET value is higher than the one obtained in the context of the wild type with Mg^2+^ ions (*E*_FRET_ ∼ 0.37), consistent with the P2 stem being in closer proximity to the P1 stem in the absence of the loop–loop interaction. The presence of DAP was found to further reduce the FRET value to ∼0.37 (Figure 5B, bottom panel), suggesting that ligand binding caused the L2 mutant to adopt a conformation similar to the wild-type F state (Figure 5A, middle panel). Together, these results are consistent with the P2 stem being reorganized both upon the formation of the intermediate and folded structures.

To gather additional information about the folding pathway of single *add* aptamers, analysis of smFRET time traces obtained using the P1–P2 vector was performed in the context of the wild-type aptamer at a low Mg^2+^ ions concentration (400 μM). Aptamers were found to dynamically exchange between the U and F states (Figure 5C), indicating that single aptamers transiently sample both conformers. The absence of the I state is consistent with a rapid exchange occurring with the F state (27). Similarly, the intermediate conformer was not detected when performing smFRET analysis at various Mg^2+^ ion concentrations (Supplementary Figure S10). However, when smFRET measurements were performed by replacing Mg^2+^ with Na^+^ ions, single aptamers were observed to transit between the U, I and F states (Figure 5D), indicating that the I state is stabilized by the absence of Mg^2+^ ions.

### smFRET analysis of the core region of the *add* aptamer.

So far, our smFRET analysis of the *add* aptamer suggested that the global folding is dictated by the binding of both Mg^2+^ ions and metabolite. Importantly, while smFRET measurements monitoring interhelical distances yield invaluable data about the global folding of the aptamer, they do not offer structural insights about the aptamer core region that is involved in metabolite recognition.

**Figure 6.**
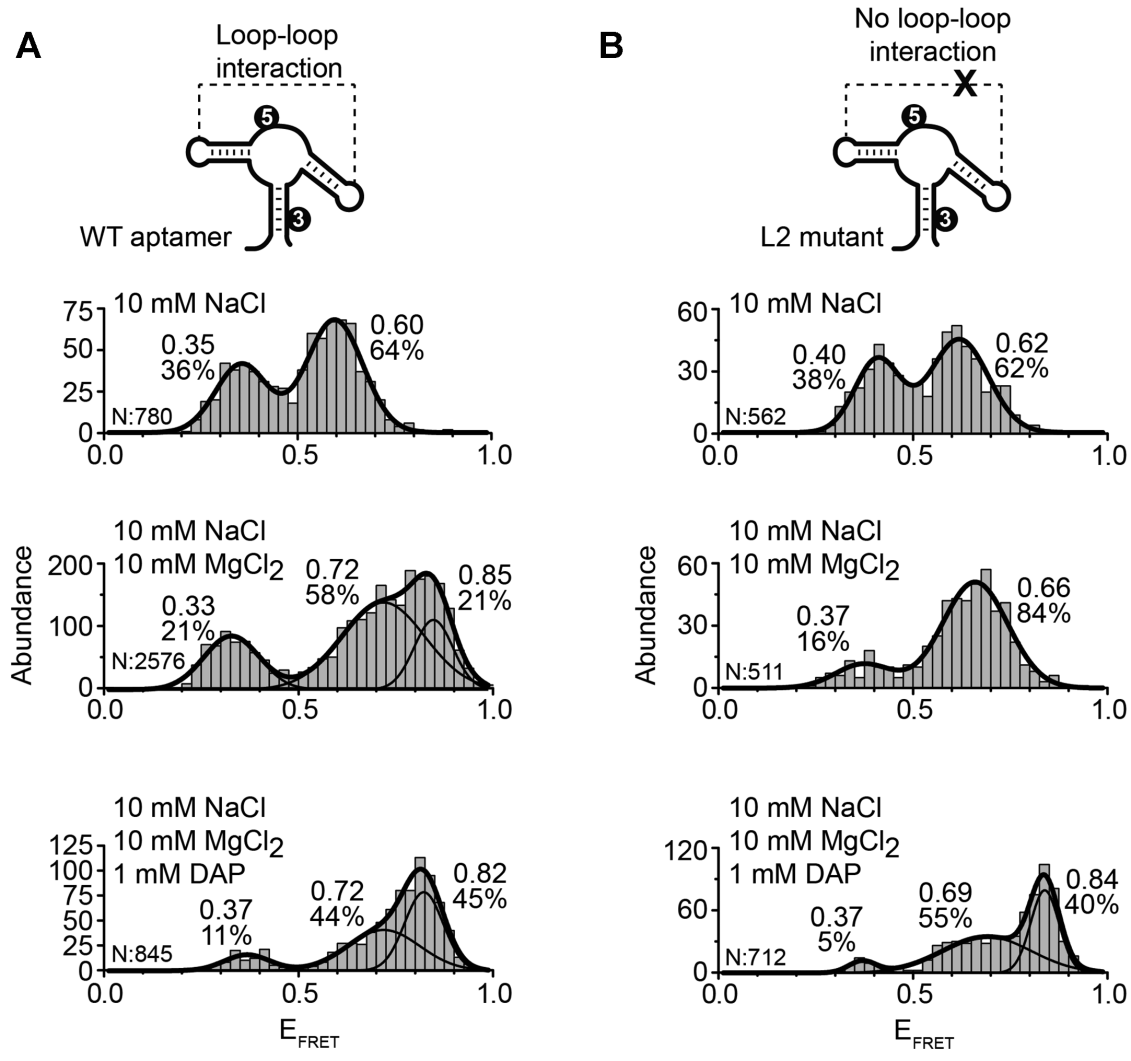
smFRET analysis for the J2/3-P1 core region of the adenine aptamer. (**A**, **B**) smFRET experiments performed for the wild-type (A) and the L2 mutant (B) aptamers. The cartoon represents the dual-labeled aptamer used in each case. Cy3 (3) and Cy5 (5) fluorophores are indicated. Population histograms are shown in presence of 10 mM NaCl (top panel), 10 mM NaCl and 10 mM MgCl_2_ (middle panel) and 10 mM NaCl, 10 mM MgCl_2_ and 1 mM DAP (lower panel). E_FRET_ values determined by fitting analysis for each population are indicated. The number of analyzed molecules (N) and the proportion of each analyzed population are indicated.

To study the folding of the core region, Cy3 and Cy5 fluorophores were introduced in the P1 stem and in the J2/3 single-strand region, respectively (Figure 6A, see scheme). The presence of the Cy5 dye within the J2/3 region could potentially perturb ligand recognition as it is in the vicinity of the binding site. To avoid this, the Cy5 dye was positioned at the residue 42 (Figure 1B) since it can accommodate any residue apart from adenine (21,36,38). This position can also tolerate a 2-aminopurine fluorophore without perturbing the ligand binding activity (38,41). Lastly, the nucleotide 42 was not observed to interact with the remaining part of the aptamer in the folded states (24,25), suggesting that the linker used for Cy5 introduction should not interfere with aptamer folding.

In conditions allowing the U state (NaCl), smFRET measurements revealed two populations (*E*_FRET_ ∼0.35 and ∼0.60) (Figure 6A, top panel), suggesting that the core region in the unfolded conformation transits between two different structures. Since mostly the U state is folded in the presence of Na^+^ ions (Figure 2A, top panel), it suggests that both core conformations fold within the U state. When these experiments were repeated in the presence of Mg^2+^ ions to allow the formation of the F state, three distinct core conformations were observed (*E*_FRET_ ∼0.33, ∼0.72 and ∼0.85) (Figure 6A, middle panel). Based on the similar FRET values observed across NaCl and Mg^2+^ conditions, we hypothesized that aptamers showing low FRET efficiencies in NaCl (*E*_FRET_ ∼ 0.35) and Mg^2+^ ions (*E*_FRET_ ∼ 0.33) correspond to the same unfolded core conformation. As a result, it suggests that the high-FRET population observed in NaCl (*E*_FRET_ ∼ 0.60) may sample two different conformations in Mg^2+^ ions (*E*_FRET_ ∼0.72 and ∼0.85). Lastly, in the presence of DAP where the F* state is favored, we observed similar core conformations occurring (*E*_FRET_ ∼0.37, ∼0.72 and ∼0.82) (Figure 6A, lower panel). However, we found that in these conditions the high FRET population (*E*_FRET_ ∼ 0.82) was the predominant conformation, consistent with the structure of the core becoming more compact upon ligand binding (24,25).

When monitoring the folding of the core region in the L2 mutant, aptamers were found to adopt two different core conformations in the U state (*E*_FRET_ ∼0.40 and ∼0.62) (Figure 6B, top panel). The addition of Mg^2+^ ions, which is required to produce the intermediate conformer in the L2 mutant (Figure 2B, middle panel), resulted in two core conformations (*E*_FRET_ ∼0.37 and ∼0.66) where the high FRET population was more prominent (Figure 6B, middle panel). However, the addition of DAP to obtain the F* state yielded three core conformations (*E*_FRET_ ∼0.37, ∼0.69 and ∼0.84) (Figure 6B, bottom panel), which is similar to what we obtained with the wild type (Figure 6A, bottom panel). Because the high FRET population (*E*_FRET_ ∼ 0.84) is strictly obtained with DAP, it indicates that this population represents ligand-bound aptamers as observed for the wild-type sequence (Figure 6A, bottom panel).

Together, our smFRET analysis of the core region suggests that it mainly adopts two conformations showing high FRET values (*E*_FRET_ ∼0.72 and ∼0.85) upon the formation of the loop–loop interaction in the F and F* states and that their relative contributions are modulated by ligand binding.

### Folding of the aptamer core region monitored by SHAPE assays.

**Figure 7.**
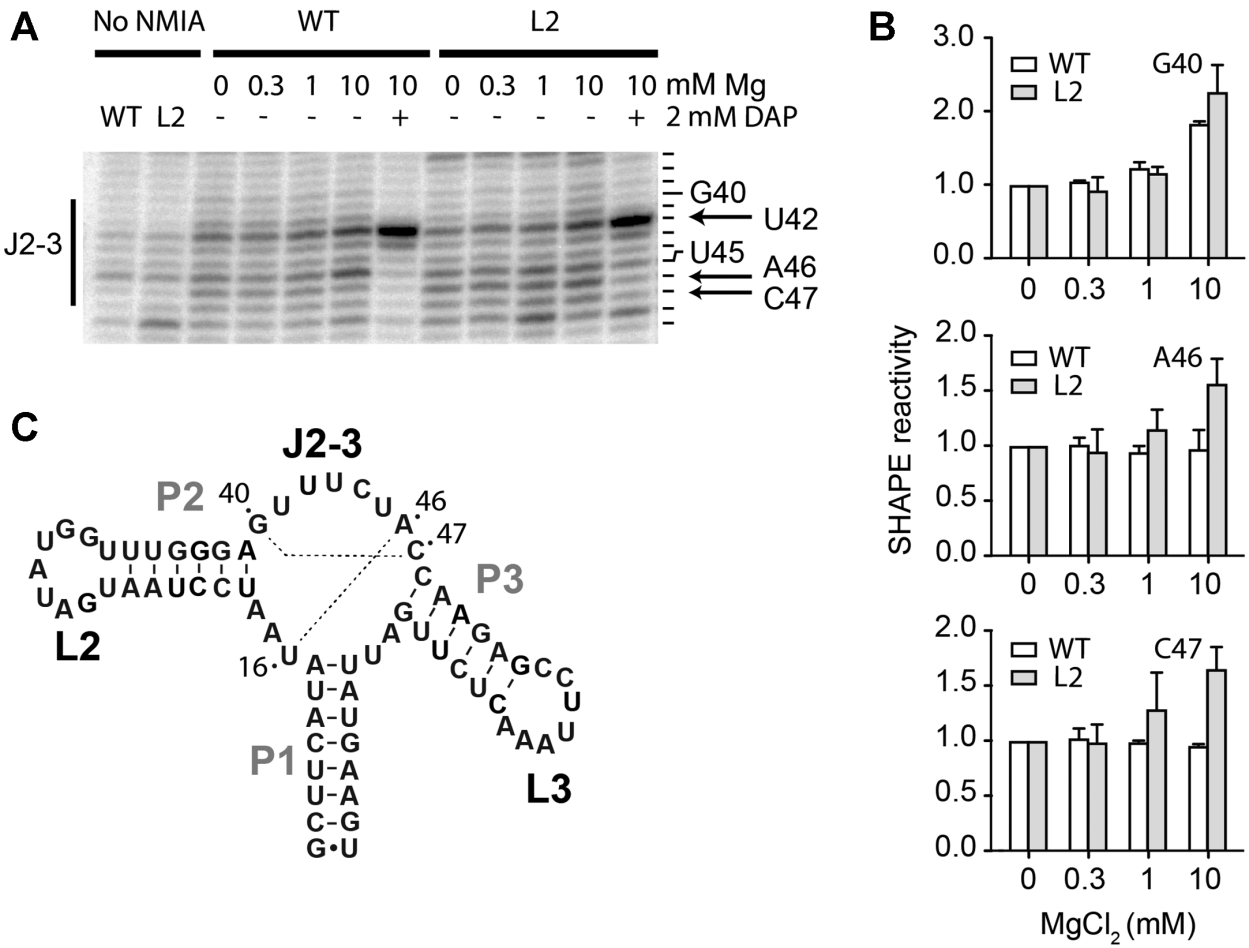
SHAPE analysis of the adenine aptamer. (**A**) SHAPE experiments performed for the wild-type (WT) and the L2 mutant (L2) aptamers. Reactions were done using various concentrations of magnesium ions (0, 0.3, 1 and 10 mM) in the absence or presence of 2 mM DAP. Control experiments were performed for both the wild type and the L2 mutant in which the NMIA reagent was replaced with DMSO. Only the region J2–3 of the aptamer is shown. The reacting nucleotides are shown on the right of the gel and the arrow shows position A46. The complete gel is shown in the Supplementary Figure S11. (**B**) Quantification of SHAPE data obtained at various magnesium ions concentrations for positions G40, A46 and C47. The average and the standard errors are shown. (**C**) Secondary structure and sequence of the add aptamer showing the presence of Watson-Crick base pairs occurring within the core domain of the aptamer (dotted lines).

According to smFRET analysis, while the wild-type aptamer allows the loop–loop interaction in the presence of Mg^2+^ ions, the L2 mutant adopts the intermediate state in which the loop–loop structure is not yet folded (Figures 2A and 2B, compare middle panels). To get more structural information about the core domain in the context of the intermediate state, selective 2′-hydroxyl acylation analyzed by primer extension (SHAPE) analysis (55) was performed. The SHAPE technique allows to monitor the accessibility of nucleotide residues and to determine the local conformation of RNA structures. SHAPE analysis was first performed on the natural sequence of the *add* aptamer (see Supplementary Table S3 for the sequence) using a range of Mg^2+^ ions concentrations (Figure 7A and Supplementary Figure S11). In the absence of Mg^2+^ ions, SHAPE modification was detected for most residues located in L2 and L3 single stranded regions, as expected from previous studies (32,36). However, while the addition of Mg^2+^ ions resulted in a small protection of L2 loop residues, a stronger effect was observed for the L3 loop, which is consistent with the formation of the loop–loop interaction upon Mg^2+^ ions binding. While the addition of DAP resulted in the protection of several regions within the aptamer domain, the residue U42 was found to be highly reactive (Figure 7A), as expected from its high solvent exposition in the crystal structure (24). When these experiments were repeated in the context of the L2 mutant, a similar SHAPE modification pattern was obtained (Figure 7A and Supplementary Figure S11). However, no protection of L2 and L3 residues was detected in the presence of Mg^2+^ ions, consistent with the absence of the loop–loop interaction in this context (Figure 2B, middle panel). Furthermore, higher SHAPE reactivities were observed for residues involved in the P2 stem across magnesium concentrations, suggesting that L2 mutations may perturb the stability of the P2 stem.

We next analyzed the SHAPE reactivity of the aptamer core region in the L2 aptamer mutant as we suspected that it could reveal key information about the folding of the intermediate conformer. When compared to the wild-type molecule, a higher reactivity for residues A46 and C47 was observed in the L2 mutant in 10 mM Mg^2+^ ions (Figure 7A, see arrows), suggesting that the local structure of both residues is different in the L2 mutant than in the wild-type structure (Figure 7B). Such higher reactivities for A46 and C47 in the L2 mutant suggest that the U16–A46 and G40–C47 base pairs are not yet formed in the context of the intermediate structure. In support to this, we observed a similar reactivity profile for G40 (Figure 7B). Although no such profile was monitored for U16 (Supplementary Figure S11), our SHAPE data still suggest that base pairs suggesting that U16–A46 and G40–C47 are not yet formed in the intermediate structure as U16 could be involved in other interactions in this context. When performing SHAPE probing in the presence of DAP, protections were observed for A46 and C47 (Figure 7A) indicating that the local structure of A46 and C47 are similar to the wild type in the ligand-bound structure.

## DISCUSSION

The intrinsic flexibility of RNA molecules is crucial for the adoption of biologically relevant structures (1,2,4,56). For example, the folding of large RNA molecules—such as group I/II introns and RNase P—was shown to contain multiple possible folding pathways involving several long-lived intermediates (1,57). In the case of the group I intron, it was found that an intermediate conformer along the folding pathway corresponds to a misfolded state that slows down the kinetics of RNA folding (58,59). The group II intron was also shown to contain an obligate intermediate along the folding pathway (60), whose formation is rate limiting for ribozyme activity. Clearly, the presence of discrete intermediate states along RNA folding pathways appears to be an integral part of RNA-based biological activity and regulation.

An interesting feature observed from crystal structures of purine-sensing riboswitch aptamers is that the bound ligand is totally surrounded by RNA (24,25), suggesting that ligand recognition is achieved through a partially folded aptamer exhibiting a less compact conformation. Such a partially folded conformer—the intermediate state—was previously observed to be stabilized upon ligand binding (27), consistent with a key role in riboswitch regulation. However, due to the relatively low contribution of the I state and its fast interconversion with the U and F conformers (27), the structural characterization of the I conformer has remained difficult. The present work demonstrated using smFRET that by balancing the concentration of monovalent ions that promote aptamer folding and the concentration of urea molecules acting as unfolding agents, it is possible to shift the folding equilibrium towards the I state. At concentrations of urea (2M) and Na^+^ (250 mM), where no U state is present (Supplementary Figure 6B), the I state became the predominant species (∼78%) and exhibited a FRET efficiency value of ∼0.46. This value is similar to that observed for the low-populated I state using the same P2–P3 vector at moderate concentrations of Mg^2+^ ions (27) (Figure 2B) and confirms that aptamer folding takes place via a discrete intermediate conformation lacking the loop–loop interaction.

**Figure 8.**
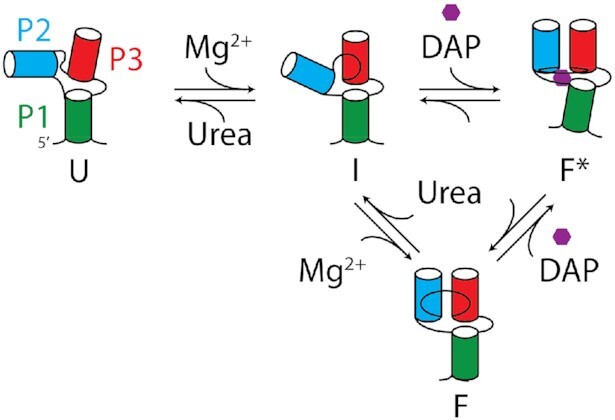
Folding pathway of the add adenine aptamer. The folding pathway is constituted by four different conformers which are the unfolded (U), the intermediate (I), the ligand-free folded (F) and the ligand-bound folded (F*) states. The P1, P2 and P3 stems have been labeled in green, blue and red, respectively. Our results are consistent with the U state being mostly unstructured but in which the P1–P3 coaxial stacking is probably already folded. The binding of magnesium ions promotes the folding of the I state that is characterized by the global repositioning of the P2 stem relatively to the P1–P3 helical stack. SHAPE data suggest that the core region of the I state does not yet contain the presence of the U16–A46 base pair. Further restructuration of the aptamer leading to the formation of the loop–loop interaction is indicative of the F state. Lastly, an interconversion between the F and F* states is also possible upon ligand binding in which the P1 stem is repositioned relatively to the rest of the aptamer. Our results are also consistent with ligand recognition performed by the I state that directly leads to the F* state (27).

The *add* aptamers undergo structural changes as they transit through U, I, F and F* states (Figure 8). While the U state adopts a relatively extended conformation, the folding into the I state mainly consists in the P2 stem being positioned closer to the P3 stem without being close enough to allow the formation of the L2–L3 loop–loop transition (Figure 8). Since very small changes are observed when monitoring the P1–P3 FRET vector (Figure 4), it suggests that the stacking interaction is already folded in the context of the U state. Our results indicate that aptamers transiting from the I to the F state show an additional decrease of the P2–P3 distance that is most probably important for the formation of the loop–loop interaction. In the event that aptamers transit from the F to the F* state due to ligand binding, our smFRET data suggest that the P1 stem is repositioned relatively to the rest of the aptamer. Interestingly, since these results showed that ligand recognition does not significantly alter the P2–P3 and P1–P3 distances (Figure 2A, lower panel and Figure 4A, lower panel), it suggests that the ligand-dependent change in P1–P2 FRET efficiency does not result from a variation of the distance between both dyes. Rather, the FRET change could be due to an axial rotation of the P1 helix, as previously observed for the SAM riboswitch (51), which could be caused by the reorganization of the junction core domain upon ligand binding (20). This is in very good agreement with XFEL data showing that the P1 stem is differently positioned relatively to P2 and P3 stems in ligand-bound structures (15). Our data is also in good agreement with a previous study of the guanine aptamer showing that the P2 stem is highly dynamic between two orientations (43). Together, these smFRET data indicate that the intermediate conformer represents a partially folded structure that is adopted prior to the formation of the F state, thus providing the required scaffold to perform ligand binding (27). This is supported by SHAPE results indicating that base pairs U16–A46 and G40–C47 of the *add* aptamer are not yet formed in the intermediate conformer (Figure 7), consistent with the core domain of the aptamer being partially folded in this context.

smFRET analysis of the aptamer core region revealed that at least three different structures are sampled (Figure 6). These results show an important flexibility of the core region of the *add* aptamer that is most likely required to allow ligand accessibility to the binding site. In the presence of 10 mM MgCl_2_, where the L2–L3 interaction is formed (Figure 6A, middle panel), the core region is mostly found in two high FRET conformations (*E*_FRET_ ∼0.72 and ∼0.85) that can be modulated by ligand binding (Figure 6A, lower panel). These results suggest that the *add* aptamer performs ligand recognition using a conformation in which the L2–L3 interaction is formed and where the core region is relatively structurally dynamic, as previously proposed (39). The presence of the L2–L3 interaction may restrict the conformational search space that the core region may sample and direct the folding toward productive conformations for ligand binding. The highest FRET state that is achieved upon DAP binding is reminiscent of the crystal structures (24,25) showing a relatively compact aptamer core region where the J2/3 strand is interacting with the bound ligand and with the P1 stem. Interestingly, the core region showed the most heterogeneous population histogram in presence of magnesium (Figure 6A middle panel) consistent with the increased flexibility necessary for ligand binding. Furthermore, smFRET data obtained in the presence of ligand suggest that the core region of the wild type and L2 mutant exhibit a similar conformation (*E*_FRET_ ∼0.82 versus ∼0.84, respectively). However, our SHAPE analysis (Figure 7) also show that local structural differences in the core as the residue A46 is more accessible in the L2 mutant compared to the wild type in presence of magnesium. Our analysis of the core region in the *add* aptamer depicts a mostly dynamic domain that is modulated by ligand binding.

Both adenine and guanine-sensing aptamers exhibit relatively similar structures (24,25). It seems that both RNA species share similar folding strategies as they both show high structural flexibility of the P2 helical domain (43) (Figure 5), which may be useful to perform a conformational search prior to the formation of the loop–loop interaction (27,42,45). However, while an intermediate conformer was observed for the adenine aptamer and its role in riboswitch function is demonstrated here, no such conformer was detected in the case of the guanine aptamer (43). Given that both aptamers show key differences in their junction domain, such as residues 42 and 68 which are involved in determining ligand binding specificity (20,34,35), it is possible that the interplay between folding and ligand recognition follows a different mechanism in both aptamers. Interestingly, smFRET analysis of the guanine aptamer also revealed that the P1–P2 vector shows an increase in FRET upon the binding of magnesium ions or ligand (43), which is in contrast to what we observed for the adenine riboswitch (Figure 5A). Therefore, it is possible that the P1–P2 distance increase in the guanine aptamer either reflects a different structural change or that the inter-dye distance is differently affected in both aptamers. Further mutational studies will be required to determine if the presence of the intermediate in the adenine aptamer is dependent on the identity of positions 42 and 68.

While it was previously observed for the guanine riboswitch that mutations preventing the formation of a single base pair within the loop–loop structure do not inhibit ligand binding (40), the replacement of wild-type loops with UUCG tetraloops completely abolished metabolite sensing (25). These results suggest that some degree of interaction is required between the loops for the aptamer to efficiently stabilize the guanine-bound complex. smFRET data indicate that the partial or complete disruption of all base pairs involved in the loop–loop interaction can be achieved while still retaining ligand binding (L2, L3 and G31C/G32C mutants), consistent with the remaining hydrogen bond interactions between both loops being sufficient to support ligand binding. Importantly, given that loop mutants do not adopt the F state in the absence of the metabolite, it suggests that the loop–loop structure is folded through an induced-fit mechanism upon ligand binding. An induced-fit mechanism was previously proposed for the guanine riboswitch where the aptamer core domain was rearranged prior to ligand binding (39). In addition to the lysine and preQ1 riboswitches (61–63), the induced-fit mechanism has been shown to also be employed by the TPP-sensing riboswitch, which is also organized around a three-way junction (64). Further experiments will be required to determine whether three-way junction motifs are important for induced-fit mechanisms of RNA molecules.

Finally, the folding pathway of the adenine aptamer was previously characterized using force microscopy where it was found that the formation of the P1 stem was not required prior to metabolite binding (65). However, since the study was performed using the transcriptionally-regulating *pbuE* riboswitch, the conclusions they reached may not be applicable to the *add* aptamer, which is operating at the translational level. Indeed, as opposed to the *pbuE* riboswitch, the *add* riboswitch was previously shown to perform ligand binding post-transcriptionally (66) and that at least 4 bp of the P1 stem are required for efficient binding (34). These results are consistent with transcriptionally- and translationally-regulating riboswitches exhibiting different metabolite binding mechanisms (66), which would be important for metabolite recognition to be performed cotranscriptionally and post-transcriptionally, respectively. The folding pathway presented here is representative of a post-transcriptional binding of the ligand to the *add* aptamer and suggests a hierarchical process involving the role of an intermediate conformer along the folding pathway. The results presented here could be applied to study more complex riboswitches or RNA-based regulation mechanisms.

## DATA AVAILABILITY

The data that support the findings of this study are available from the corresponding author, D.A.L., upon reasonable request.

## Supplementary Material

gkab307_Supplemental_Files

## Notes

Present address: Paul A. Dalgarno, Institute of Biological Chemistry, Biophysics, and Bioengineering, School of Engineering and Physical Sciences, Heriot-Watt University, Edinburgh EH14 4AS, UK.
