# Supplementary material for: A structural intermediate pre-organizes the add adenine riboswitch for ligand recognition
Source: Nucleic Acids Res. 2021 May 8;49(10):5891–904. doi: 10.1093/nar/gkab307 (PMC8191784; doi:10.1093/nar/gkab307)
Supplement: gkab307_Supplemental_Files [file gkab307_supplemental_files.zip › St-Pierre.NAR-Supp.2020 accepted revision.pdf]

A structural intermediate pre-organizes the *add* adenine riboswitch for ligand recognition

Patrick St-Pierre[1], Euan Shaw[2], Samuel Jacques[1], Paul A. Dalgarno[3], Cibran Perez-Gonzalez[4], Frédéric Picard-Jean[1], J. Carlos Penedo[*2,4] and D.A. Lafontaine[*1]

[1]Department of Biology, Faculty of Science, Université de Sherbrooke, Sherbrooke, Quebec, Canada, J1K 2R1.

[2]Centre of Biophotonics, School of Physics and Astronomy, University of St Andrews, St Andrews, Fife, KY16 9SS, UK

[3]Present address: Institute of Biological Chemistry, Biophysics, and Bioengineering, School of Engineering and Physical Sciences, Heriot-Watt University, Edinburgh EH14 4AS, UK

[4]Biomedical Sciences Research Complex, University of St Andrews, St Andrews, Fife, KY16 9SS, UK

*To whom correspondence should be addressed: jcp10@st-andrews.ac.uk and daniel.lafontaine@usherbrooke.ca

Keywords: single-molecule detection, Förster resonance energy transfer (FRET), transient RNA conformations, riboswitch, urea

SUPPLEMENTARY INFORMATION

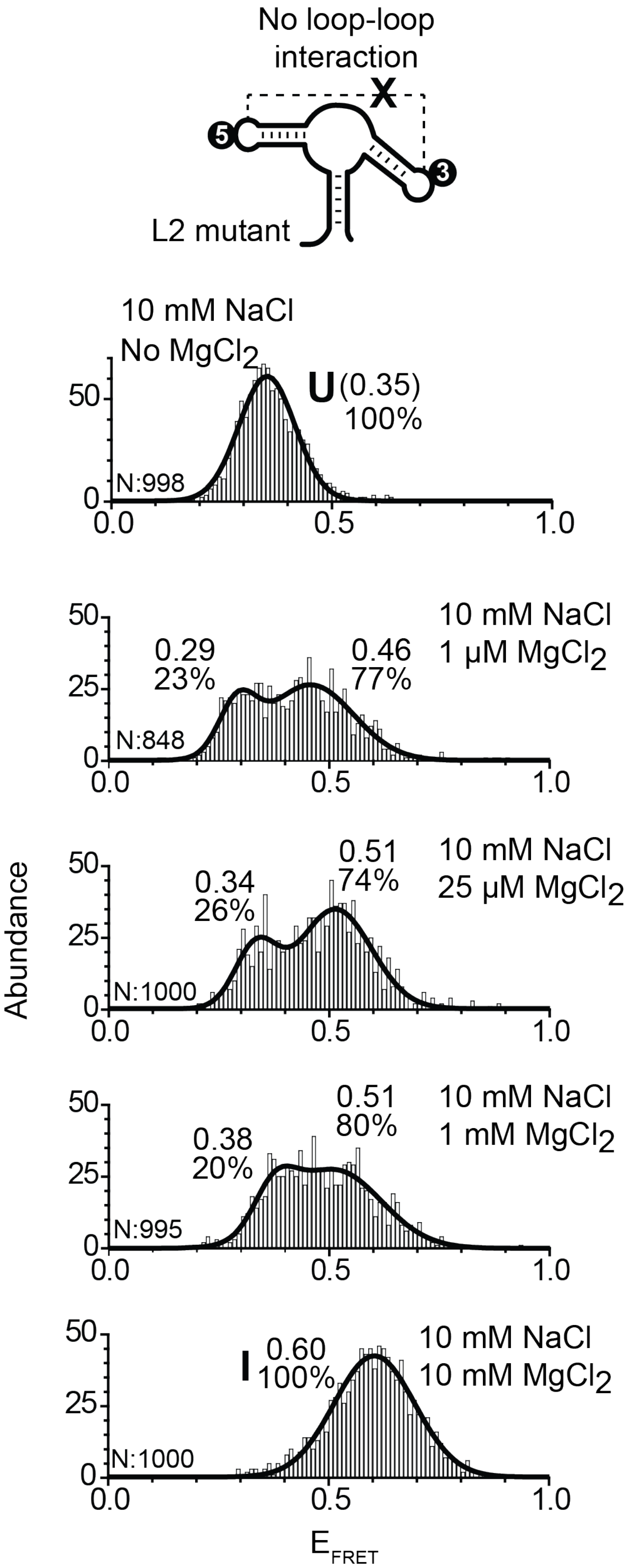

Supplementary Figure S1. smFRET histograms for the vector P2-P3 of the L2 mutant aptamer.

Population histograms were collected in presence of 10 mM NaCl. The concentration of magnesium ions is indicated in each case. The cartoon represents the dual-labeled aptamer. Cy3 (3) and Cy5 (5) fluorophores are indicated. E_{FRET} values determined by fitting analysis for each population are indicated in each case. Unfolded (U) and intermediate states (I) are shown.

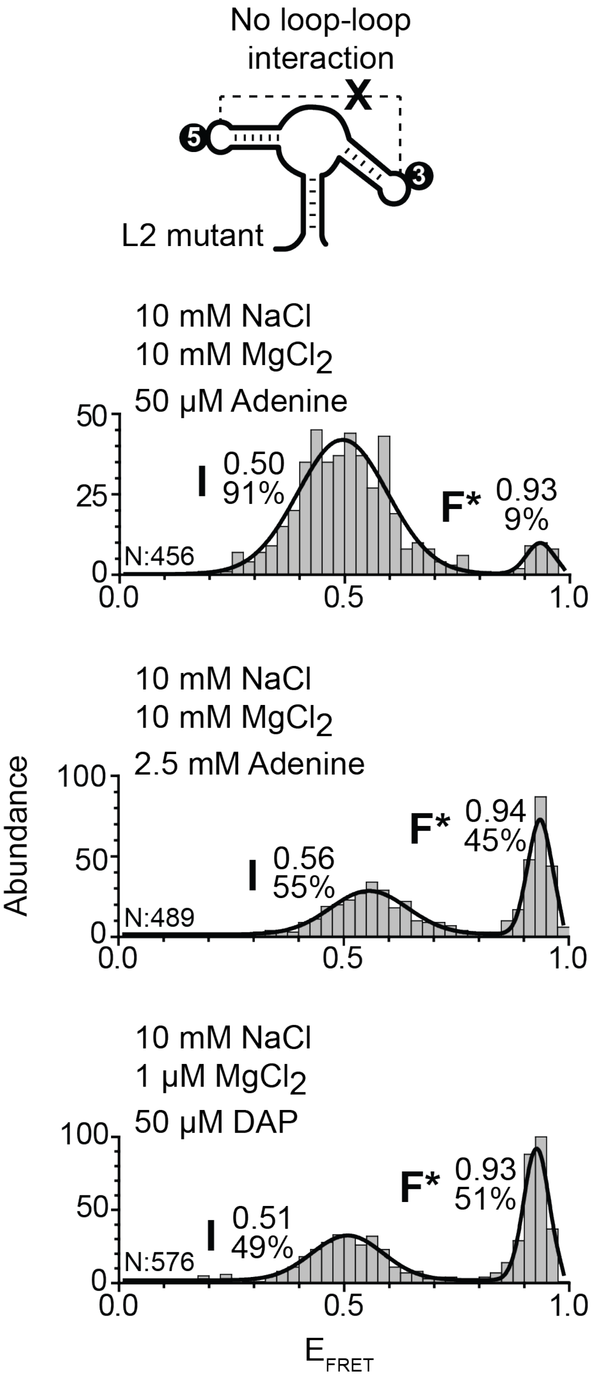

Supplementary Figure S2. smFRET control experiments using the vector P2-P3 of the L2 mutant aptamer.

Population histograms are shown in presence of 10 mM NaCl, 10 mM $MgCl_2$ and 50 µM adenine (top panel), 10 mM NaCl, 10 mM $MgCl_2$ and 2.5 mM adenine (middle panel) and 10 mM NaCl, 1 µM $MgCl_2$ and 50 µM DAP (bottom panel). The cartoon represents the dual-labeled aptamer. Cy3 (3) and Cy5 (5) fluorophores are indicated. E_{FRET} values determined by fitting analysis for each population are indicated. The intermediate (I) and the ligand-bound folded (F*) states are shown.

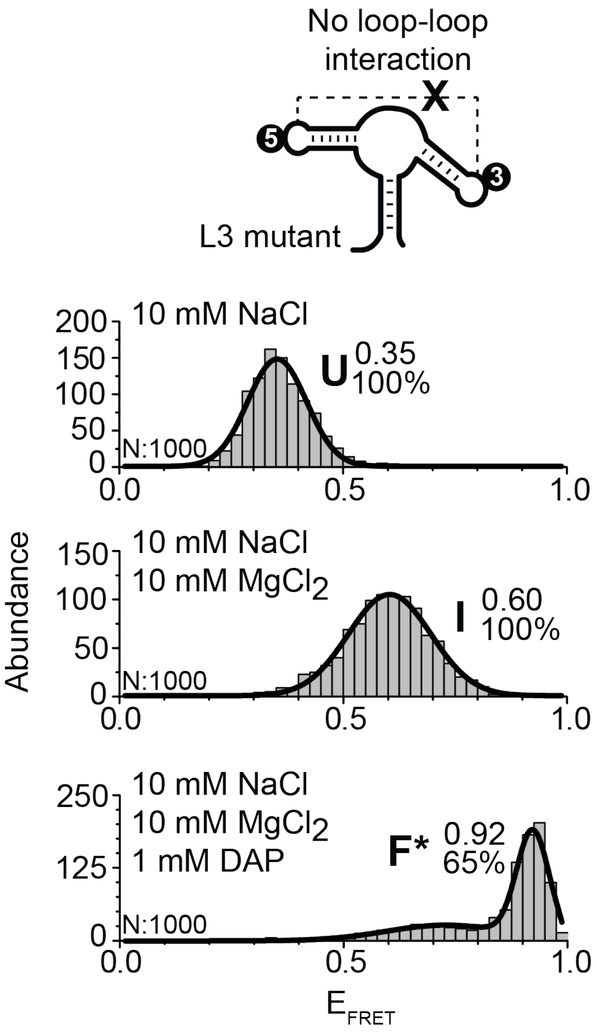

Supplementary Figure S3. smFRET analysis for the vector P2-P3 of the L3 mutant aptamer.

Population histograms are shown in presence of 10 mM NaCl (top panel), 10 mM NaCl and 10 mM $MgCl_2$ (middle panel) and 10 mM NaCl, 10 mM $MgCl_2$ and 1 mM DAP (lower panel). The cartoon represents the dual-labeled aptamer. Cy3 (3) and Cy5 (5) fluorophores are indicated. E_{FRET} values determined by fitting analysis for each population are indicated. Unfolded (U), intermediate (I) and the ligand-bound folded (F*) states are shown.

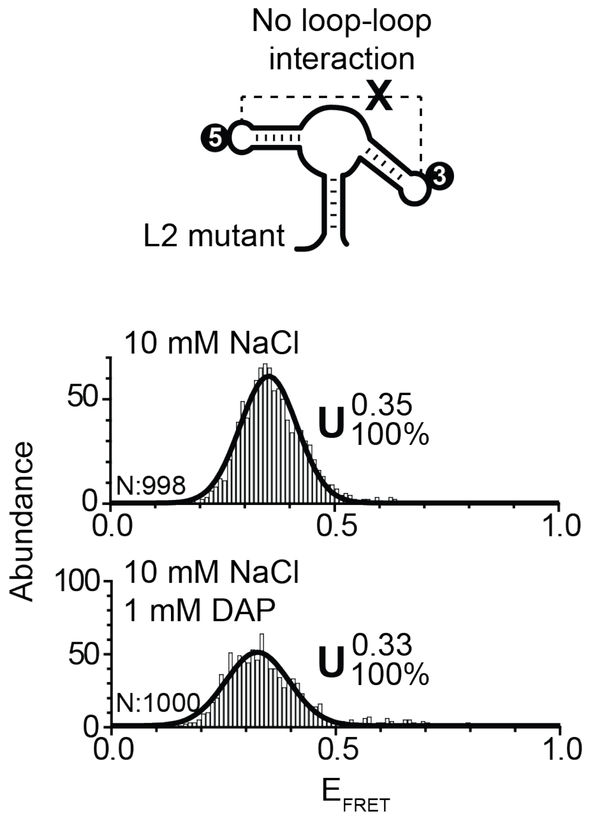

Supplementary Figure S4. smFRET analysis for the vector P2-P3 of the L2 mutant aptamer. Population histograms are shown in presence of 10 mM NaCl (top panel) and 10 mM NaCl, 1 mM DAP (lower panel). The cartoon represents the dual-labeled aptamer. Cy3 (3) and Cy5 (5) fluorophores are indicated. E_{FRET} values determined by fitting analysis for each population are indicated. The unfolded (U) state is shown.

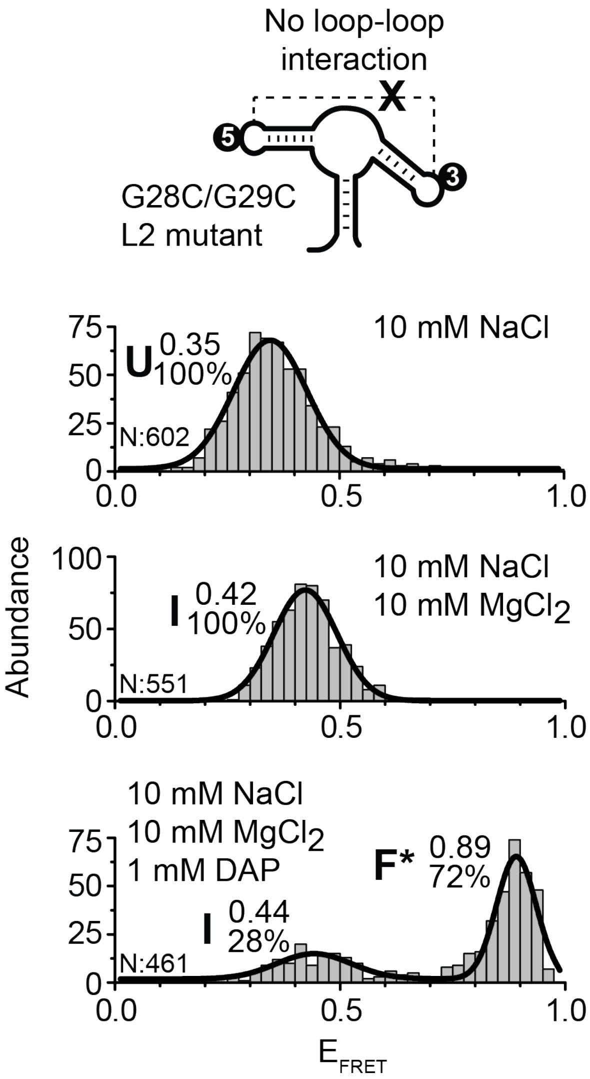

Supplementary Figure S5. smFRET analysis for the vector P2-P3 of the G28C/G29C mutant aptamer. Population histograms are shown in presence of 10 mM NaCl (top panel), 10 mM NaCl and 10 mM $MgCl_2$ (middle panel) and 10 mM NaCl, 10 mM $MgCl_2$ and 1 mM DAP (lower panel). The cartoon represents the dual-labeled aptamer. Cy3 (3) and Cy5 (5) fluorophores are indicated. E_{FRET} values determined by fitting analysis for each population are indicated. Unfolded (U), intermediate (I) and the ligand-bound folded (F*) states are shown.

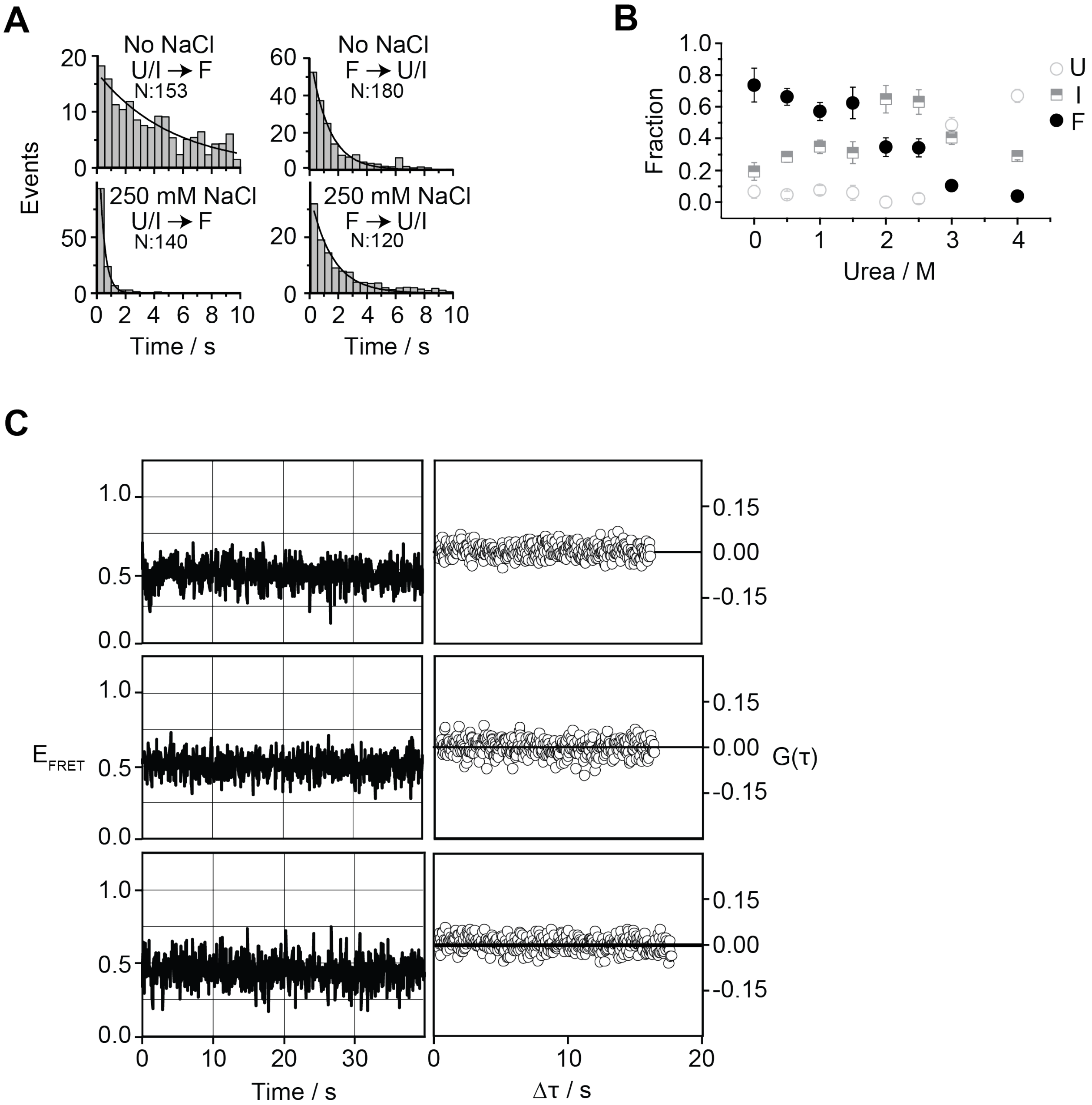

Supplementary Figure S6. smFRET analysis of the intermediate state as a function of NaCl and urea. Characterization of P2-P3 folding in the presence of Na^{+} ions and urea. **(A)** Representative single-molecule dwell-time histograms obtained for the U/I to F (left panels) and F to U/I (right panels) transitions at the indicated concentrations of Na^{+} ions. The solid line indicates the fit to a mono-exponential function from which we extracted the corresponding rates. **(B)** Relative contribution of U, I and F states as a function of urea concentration in a background of 250 mM NaCl. The relative contribution of each FRET state was obtained by fitting the single-molecule histogram FRET histogram obtained at each urea concentration to three gaussians, each corresponding to a FRET state, and calculating the percentual contribution of each gaussian to the whole histogram. **(C)** Left: single-molecule FRET trajectories obtained for the P2-P3 vector

at 2.5 M urea and 250 mM NaCl. Right: cross-correlation curves of the donor and acceptor intensity traces corresponding to the FRET trajectories shown in the left panel. The cross-correlation curves were calculated using a home-made routine implemented in Matlab using the expression: $G(\tau) = \frac{\sum(I_D(t)-\overline{I_D})(I_A(t+\tau)-\overline{I_A})}{N\sum\overline{I_D I_A}}$ where $I_D(t)$ and $I_A(t)$ are the donor and acceptor intensities at a given time point. $\overline{I_D}$ and $\overline{I_A}$ represent the mean donor and acceptor intensities over the entire single-molecule trajectory with the function normalized to the total number of data points (N). The G(τ) function compares the intensity trace of the donor at a time t and the acceptor trace at a time t+τ.

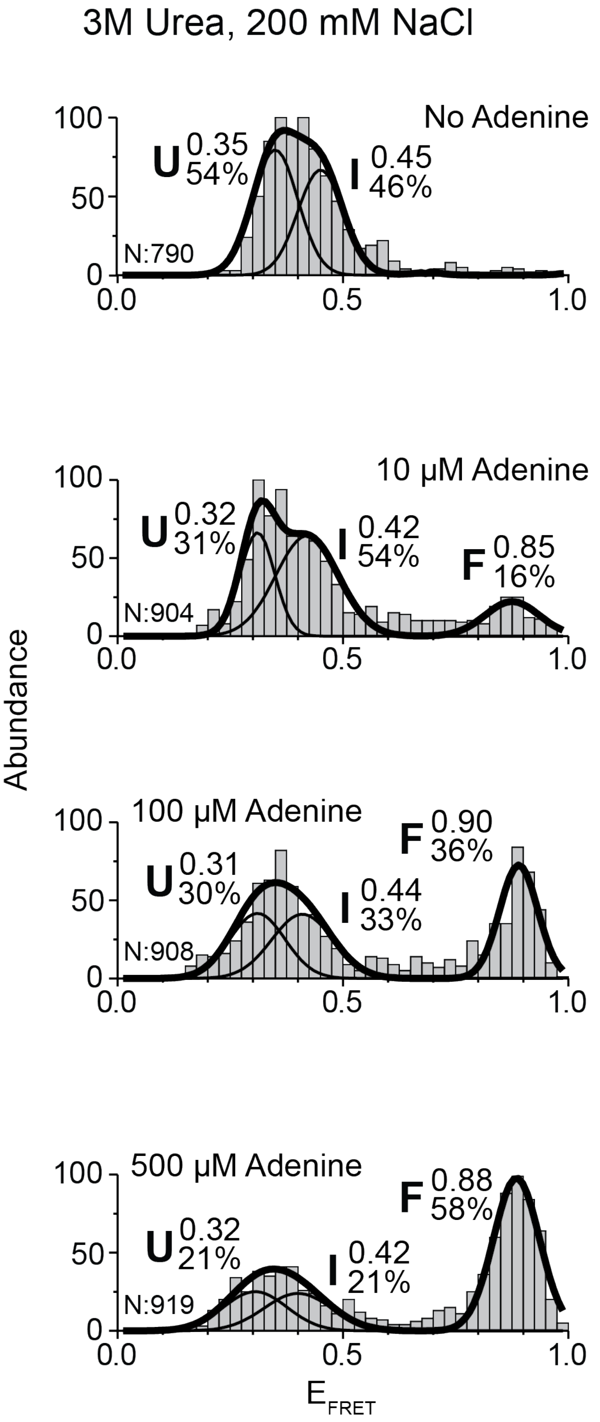

Supplementary Figure S7. smFRET histograms obtained for the vector in a background of 3M urea and 200 mM NaCl. E_{FRET} values obtained from the fitting of the experimental histogram to a sum of gaussians are shown together with the relative contribution of each population. Solid lines represent the contribution of each gaussian population and the corresponding FRET conformer is indicated. Concentrations of adenine are indicated for each experiment.

.

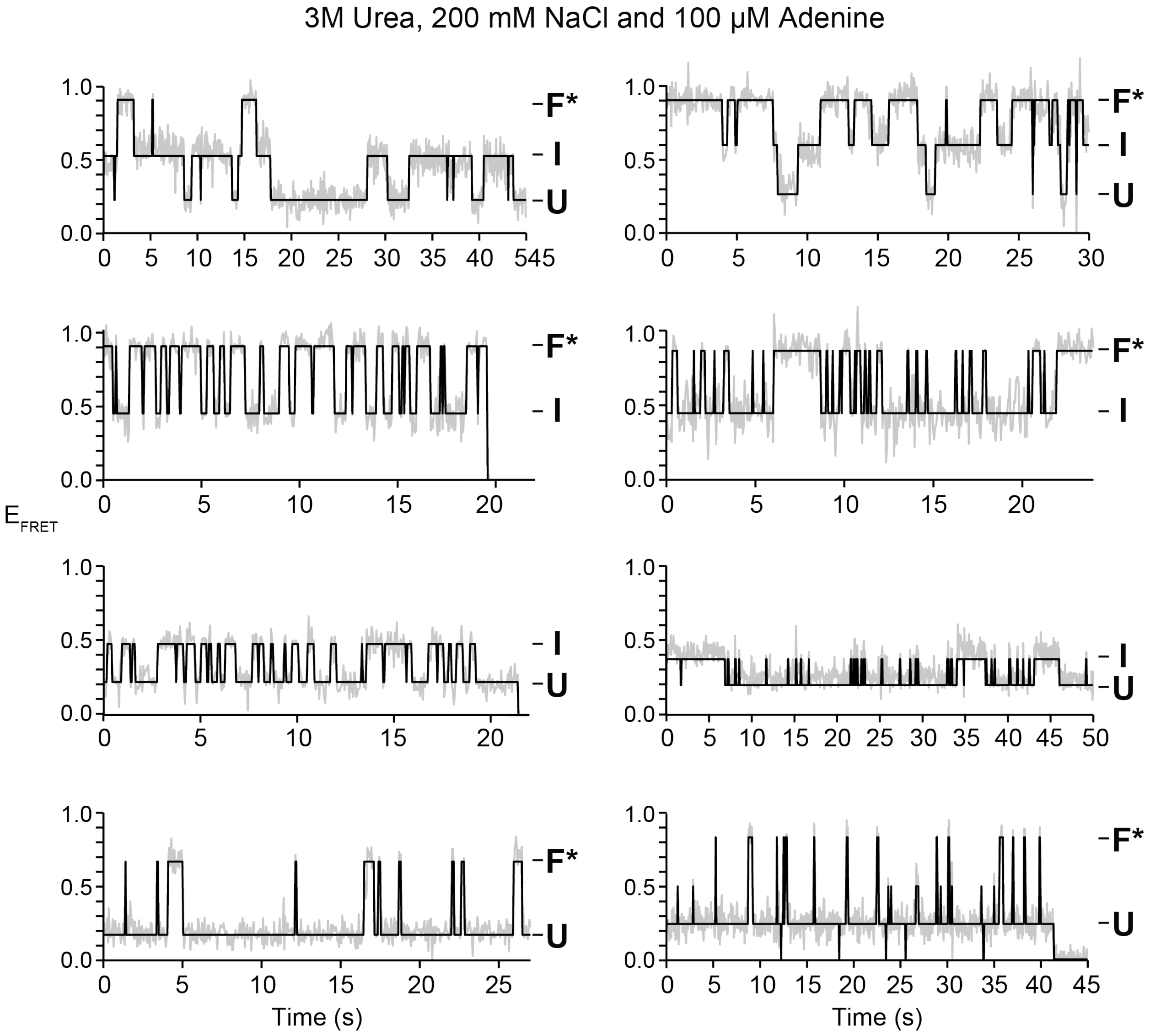

Supplementary Figure S8. Representative smFRET trajectories obtained for vector L2-L3 in a background of 3M urea and 200 mM NaCl. The solid line represents the FRET trajectory obtained from a hidden Markov modeling of the experimental trace. The corresponding single-molecule histogram for each trace is shown and shows the individual gaussians for each FRET population and the overall sum of gaussians.

.

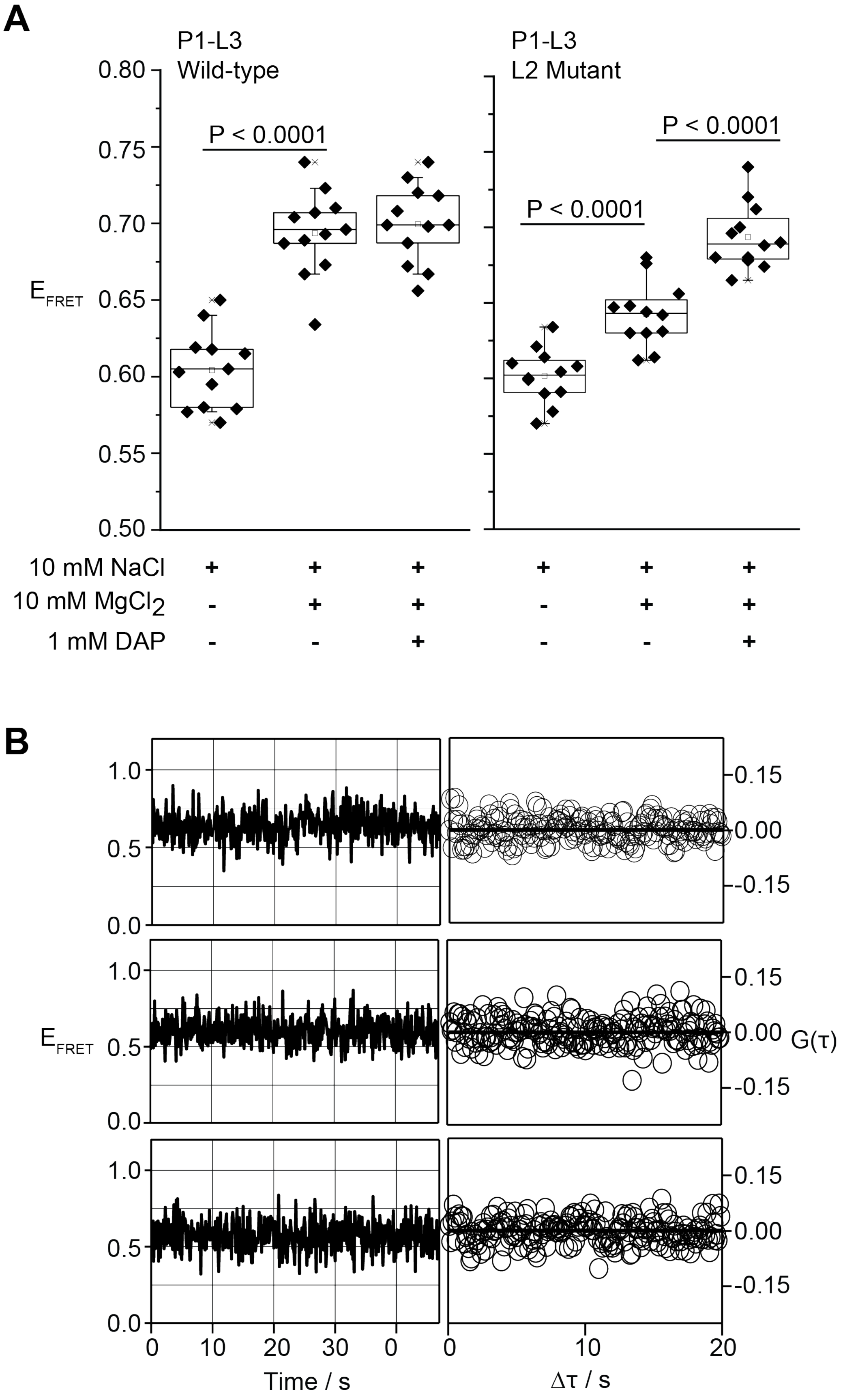
A
P1-L3
Wild-type
P1-L3
L2 Mutant
P < 0.0001
P < 0.0001
P < 0.0001
E_FRET
0.80
0.75
0.70
0.65
0.60
0.55
0.50
10 mM NaCl
10 mM MgCl2
1 mM DAP
B
E_FRET
G(τ)
Time / s
Δτ / s

Supplementary Figure S9. Comparison of the mean E_{FRET} value for the P1-L3 vector. (A) Box plot graphs were obtained for the wild-type (left panel) and the L2 mutant (right panel) (n=12 for all conditions). The extremes, upper and lower quartiles of the distribution, and the median are represented by the whiskers, box and middle lines, respectively. P-values were obtained by using an unpaired t-test are also shown. **(B)** Single-molecule FRET trajectories (left panel) obtained for the P1-L3 vector in the context of the L2 mutant at 10 mM NaCl and 10 mM $MgCl_2$. The corresponding cross-correlation curves are shown (right panel). The cross-correlation curves were calculated as indicated in the legend of Supplementary Figure S6C. The $G(\tau)$ function compares the intensity trace of the donor at a time t and the acceptor trace at a time $t+\tau$.

.

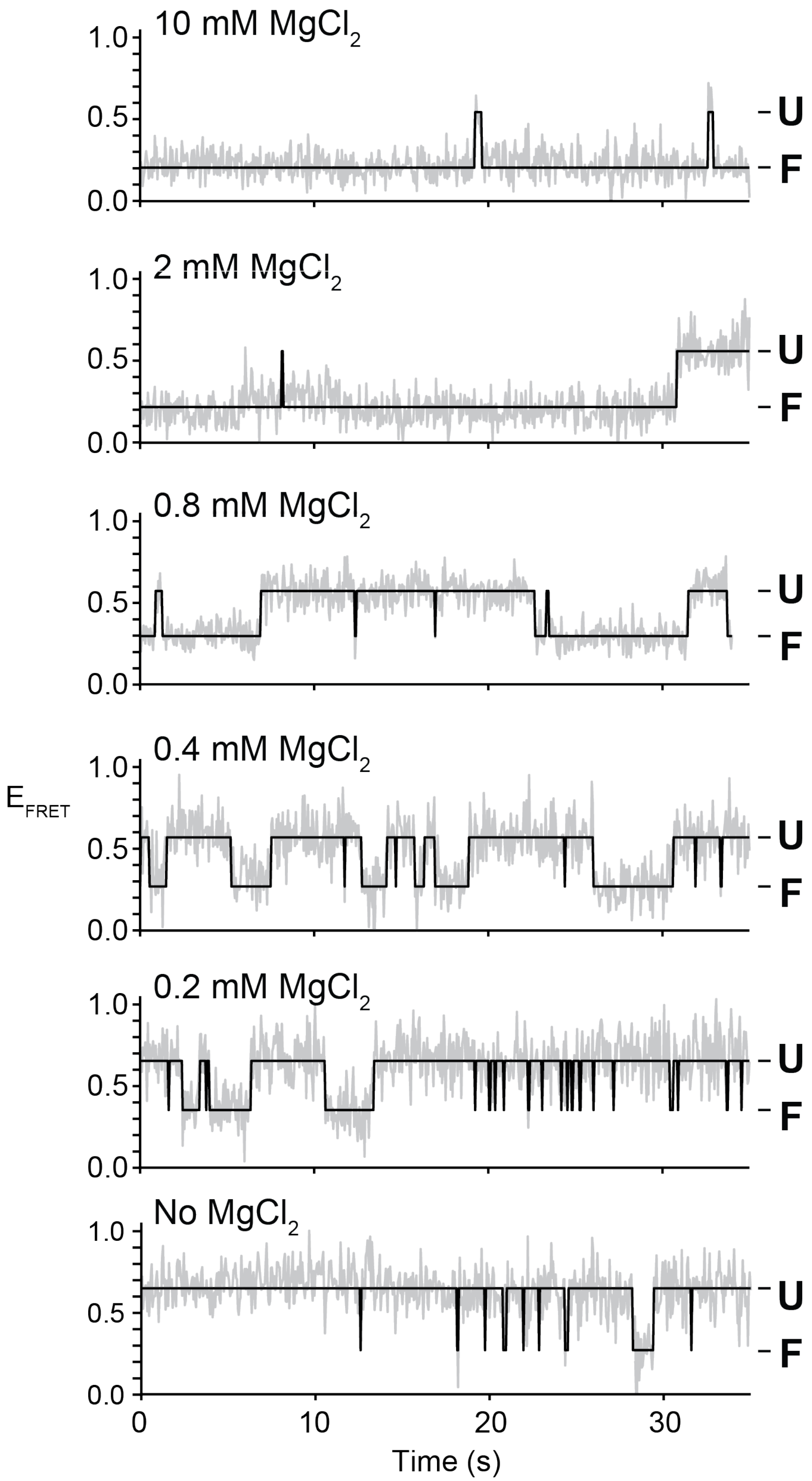

Supplementary Figure S10. smFRET time analysis for the P1-P2 vector of the adenine aptamer at various $MgCl_2$ concentrations.

smFRET time traces recorded at various $MgCl_2$ concentrations as a function of elapsed time for single aptamer molecules. The integration time is 50 ms and solid black lines represent the hidden Markov modelling of experimental traces. The assigned FRET states are indicated on the right.

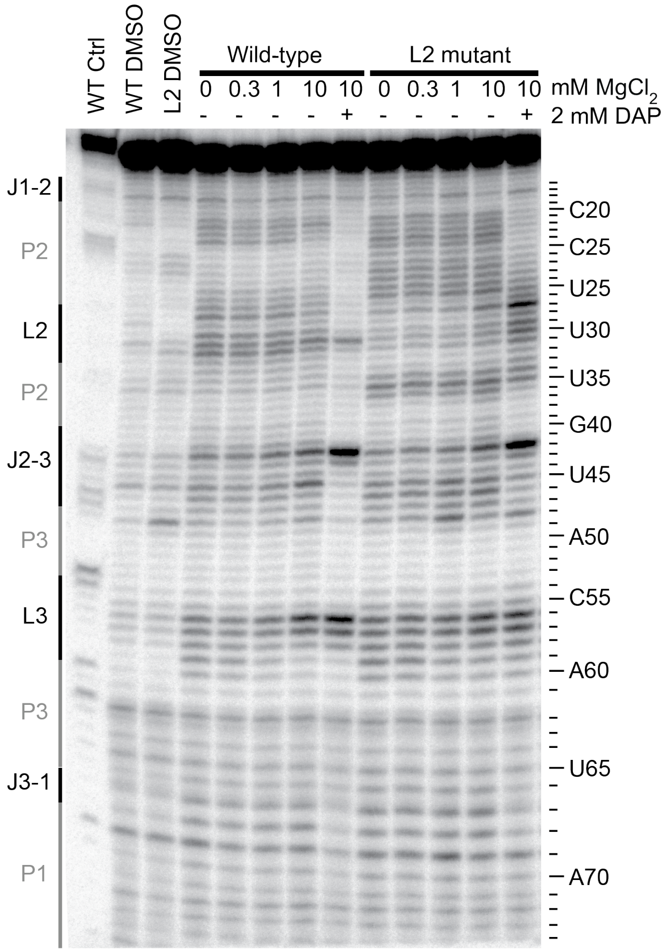

Supplementary Figure S11. SHAPE analysis of the adenine aptamer.

SHAPE experiments performed for the wild-type (WT) and the L2 mutant (L2) aptamers. Reactions were done using various concentrations of magnesium ions (0, 0.3, 1 and 10 mM) in the absence or presence of 2 mM DAP. Control experiments were performed for both the wild-type and the L2 mutant in which the NMIA reagent was replaced with DMSO. A cropped version of this gel is shown in Figure 7A.

Supplementary Table S2. Strands used to obtain smFRET constructs

Constructs	Oligonucleotides
**Vector P2-P3**	
WT 5'	Biotin-GCGCGAGCGUUGUAUAAUCCUAAUGAUAUGGUUUGGGAGU
WT 3'	PO4- UUCUACCAAGAGCCUUAAACUCUUGAUUACAACGCUCGCGC
L2 5'	Biotin-GCGCGAGCGUUGUAUAAUCCUAAUG**AAU**U**CC**UUUGGGAGU
LG28C/G29C 5'	Biotin-GCGCGAGCGUUGUAUAAUCCUAAUGUUAU**CC**UUUGGGAGU
L3 3'	PO4-UUCUACCAAGAG**GG**U**AU**AACUCUUGAUUACAACGCUCGCGC
**Vector P1-P3**	
WT 5'	Biotin-GCGCGAGCGUUGUAUAAUCCUAAUGAUAUGGUUUGGGAGU
WT 3'	PO4-UUCUACCAAGAGCCUUAAACUCUUGAUUACAACGCUCGCGC
L2 5'	Biotin-GCGCGAGCGUUGUAUAAUCCUAAUGA**AU**U**CC**UUUGGGAGU
**Vector P1-P2**	
WT 5'	Biotin-GCGCGAGCGUUGUAUAAUCCUAAUGAUAUGGUUUGGGAGU
WT 3'	PO4-UUCUACCAAGAGCCUUAAACUCUUGAUUACAAUGCUCGCGC
L2 5'	Biotin-GCGCGAGCGUUGUAUAAUCCUAAUGA**AU**U**CC**UUUGGGAGU
**Vector core-P1**	
WT 5'	Biotin-<br>GCGCGAGCGUUGUAUAAUCCUAAUGAUAUGGUUUGGGAGUUUCU<br>ACCAAGAGCCUU
WT 3'	PO4- AAACUCUUGAUUACAACGCUCGCGC
L2 5'	Biotin-<br>GCGCGAGCGUUGUAUAAUCCUAAUGA**AU**U**CC**UUUGGGAGUUUCU<br>ACCAAGAGCCUU

The positions used for labeling are identified by underlined nucleotides and mutations are shown in bold.

Supplementary Table S3. DNA and RNA sequences used to perform SHAPE assays

Molecule	Sequence
**RNA**	
WT	GCGCUUCAUAUAAUCCUAAUGAUAUGGUUUGGGAGUUUCUACCA AGAGCCUUAAACUCUUGAUUAUGAAGU**AAUUUAU**CGUCACGGUG CAUCUACCAUC
L2	GCGCUUCAUAUAAUCCUAAUGAAUUCCUUUGGGAGUUUCUACCAA GAGCCUUAAACUCUUGAUUAUGAAGU**AAUUUAU**CGUCACGGUGC AUCUACCAUC
**DNA**	
Primer	GATGGTAGATGCACCGTGACG

The underline nucleotides indicate the sequence used to hybridize the DNA primer. The positions shown in bold act as a spacer to allow better resolution of the SHAPE products on denaturing gel.
